# Nurses’ knowledge and practice on patient safety in tertiary medical college hospitals in Bangladesh: a cross-sectional study

**DOI:** 10.3389/frhs.2026.1907119

**Published:** 2026-10-16

**Authors:** Md Abdul Khalek, K. A. T. M. Ehsanul Huq, Abdulfatai Olamilekan Babaita, Chihiro Kimura, Michiko Moriyama

**Affiliations:** 1 Graduate School of Biomedical and Health Sciences, Hiroshima University, Hiroshima, Japan; 2 Nursing Advancement Division, Unnan City Hospital, Shimane, Japan

**Keywords:** Bangladesh, knowledge, nurses, patient safety, patient safety culture, practice, tertiary hospitals

## Abstract

**Background:**

Patient safety is critical to healthcare and depends on a safety culture to ensure high-quality care. A strong patient safety culture (PSC) reduces preventable patient harm, with nurses’ knowledge and practices central to delivering safe care in hospitals. Yet, evidence remains limited in tertiary medical college hospitals in Bangladesh. Therefore, this study aimed to assess nurses’ knowledge, practices, and associated factors regarding PSC in tertiary medical college hospitals in Bangladesh.

**Methods:**

A cross-sectional study was conducted from April to September 2025 in eight medical college hospitals across all eight administrative divisions in Bangladesh. Of 7,170 eligible nurses, 4,982 (valid response rate 69.5%) were analyzed using a self-administered questionnaire. Descriptive, inferential statistics and correlation were performed.

**Results:**

Participants’ mean scores were 5.44 (SD 1.82) (range: 0–10) for patient safety knowledge and 28.92 (SD 7.76) (range: 10–50) for practice. At the item level, 49.3% correctly identified adverse events, and only 9.8% knew the appropriate reporting methods. In terms of practice, 48.6% reported never using incident-reporting forms, and 26.4% reported never using structured communication tools. In bivariate analysis, participants’ education, working ward, and having in-service training were significantly associated with knowledge and younger age, hospital experience, working ward, and in-service training were significantly associated with practice. After adjusting in a mixed-effects model, professional experience (*p* < 0.018, *p* < 0.001) and in-service training (*p* < 0.001, *p* < 0.019) were significantly associated with knowledge and practice, respectively. Gender (*p* < 0.049) and diploma in nursing education (*p* < 0.027) were associated with knowledge, while age (*p* < 0.001), professional experience (*p* < 0.001), and in-service training (*p* < 0.019) were associated with practice.

**Conclusion:**

Nurses’ knowledge of patient safety increased with professional experience and in-service training; however, gender and education had negative associations. Nurses’ practices also increased with professional experience, while age and in-service training showed negative associations. Targeted, context-specific in-service training with post-training monitoring by Quality Improvement Teams is essential to improve nurses’ knowledge and practice. Strengthening patient safety reporting systems with leadership engagement, logistical support, and oversight by the government regulatory bodies may improve patient-safety practice and quality of care.

## 1 Introduction

Patient safety is a fundamental principle of healthcare and a global public health priority across diverse healthcare settings (1). The World Health Organization (WHO) defines patient safety as the prevention of errors and adverse events to patients associated with health care, encompassing the mitigation of harm from system failure and unsafe practices (2). Globally, unsafe medical care causes about 1 million deaths annually, with 75%–80% occurring in low- and middle-income countries (LMICs) (3). In LMICs, about 1 in 4 patients experience adverse events, largely driven by weak safety culture, staffing shortages, heavy workloads, and supervision gaps (4, 5). Notably, over 50% of these events are counted as preventable (6). In resource-limited Bangladesh, tertiary hospitals face increased work pressure, scarce resources, poor infrastructure, and inadequate reporting systems, which contribute to medical errors, communication failures and healthcare-associated infections (7–9).

Patient safety culture (PSC) reflects the shared values, beliefs, norms, and practices prioritizing safety, fostering a healthcare environment that minimizes harm for both patients and nurses (10). Weak PSC leads to underreporting, nurse burnout, higher preventable costs, and diminished care quality (11). Nurses, as frontline providers, play a pivotal role through their knowledge, practice and timely error reporting, directly influencing clinical outcomes and prevention of adverse events (12, 13). Strong PSC knowledge and awareness promote open reporting, speaking up, and shared learning, while a lack of it reduces care quality, weakens communication, and diminishes patient trust (14–16). Preventing errors and improving care quality rely on nurses' competence in patient safety; this directly strengthens PSC (17–19).

Assessing nurses' knowledge and practice is essential, as gaps lead to unsafe care, poor teamwork, and worse outcomes; strengthening them through education enhances overall PSC (20–22). A study in Bangladesh revealed gaps in knowledge and practice in infection prevention and control emphasizing the need for strengthening patient safety initiatives among nurses (23). Therefore, it is essential to improve nurses' ability to maintain a successful and comprehensive patient safety strategy (17, 19).

Patient safety research in LMICs, including Bangladesh, remains limited due to weak safety systems, poor reporting, and a blame culture that discourages error disclosure (24). In Bangladesh, although the National Patient Safety Strategic Plan exists, there are implementation gaps in healthcare facilities, which contribute to over 30% of hospital-acquired infections (despite limited record-keeping) (25, 26).

In public hospitals in Bangladesh, the clinical documentation was predominantly paper-based, and hospital-specific adverse-event data are not consistently available. Consequently, reliable baseline estimates of reported patient safety incidents could not be established across the study sites. National policy documents indicate ongoing efforts to introduce standardized registers and reporting tools for patient-safety events, including medication incidents, falls, surgical complications, transfusion reactions, and near misses (27). Bangladesh has only 9.9 nurses per 10,000 population, well below the WHO benchmark of 44.5 per 10,000 population (28). Inadequate staffing, administrative workload, limited nursing participation in decision-making, and gaps in leadership and governance may compromise direct patient care, teamwork, communication, supervision, and incident reporting (29, 30). These gaps underscore the need to assess patient safety from nurses' perspectives in tertiary-level hospitals.

Tertiary-level medical college hospitals require a strong PSC; assessing nurses' knowledge and practice helps identify gaps and guide improvements (31). Therefore, we developed a context-specific knowledge and practice questionnaire on patient safety to capture local challenges and enable accurate assessment and targeted improvement of nurses' safety practices. This study aimed to assess nurses' knowledge and practice and associated factors on PSC in eight tertiary-level medical college hospitals in Bangladesh.

### 1.1 Conceptual framework

The conceptual framework (Figure 1) for the study was developed based on relevant guidelines and relevant literature (32). It proposes that selected socio-demographic and professional characteristics- including age, gender, education, total professional experience, experience in current hospital, experience in current ward, working ward, in-service training and types of in-service training -may be associated with nurses' knowledge and practice regarding patient safety in public hospitals in Bangladesh. These variables were therefore examined as potential factors associated with nurses' patient-safety knowledge and practice. The framework presents hypothesized associations and does not imply causal relationships. Evidence supports socio-demographics as predictors, such as knowledge and practice of patient safety culture, event reporting, and patient safety ratings of their ward in working hospitals (33).

**Figure 1:**
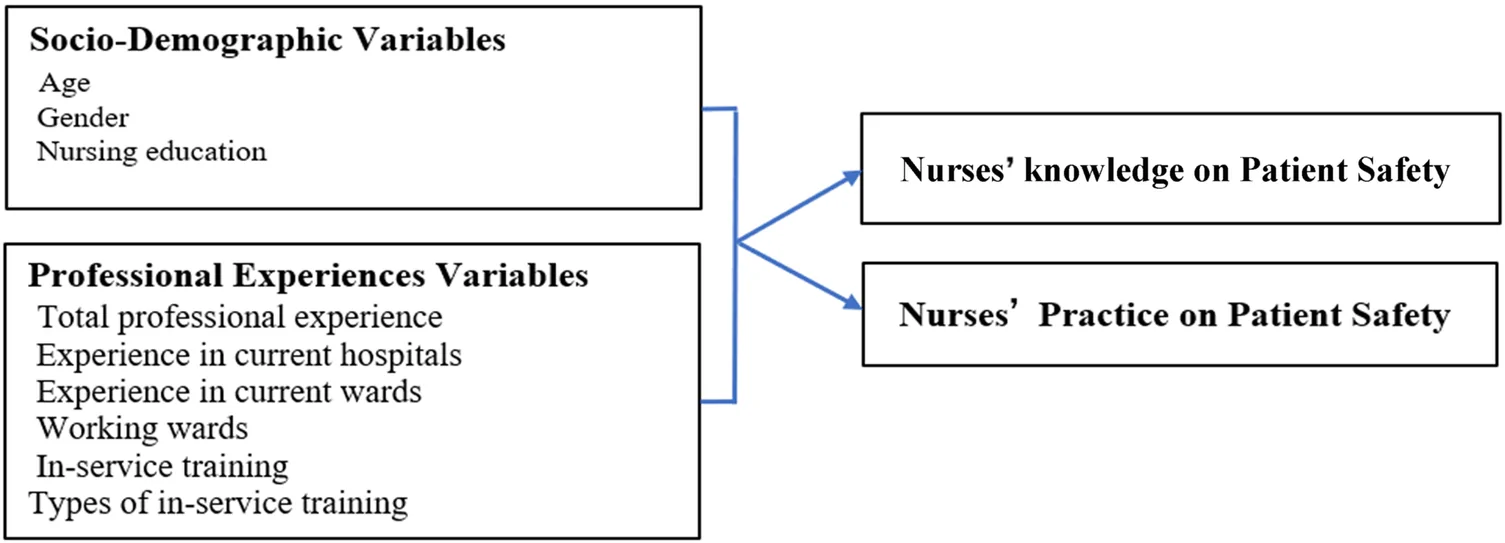
Conceptual framework.

## 2 Methods

### 2.1 Study design and settings

A cross-sectional study design was employed. We conveniently selected one tertiary-level government medical college hospital from each of the total 8 administrative divisions in Bangladesh to ensure nationwide geographical coverage. These hospitals were selected because they are major tertiary-level referral hospitals in their respective divisions, serving large and diverse patient populations. Their willingness to participate and convenient communication also facilitated conducting this study.

### 2.2 Study participants

The targeted population for this study was all eligible nurses (*n* = 8,907) from 8 participating hospitals. According to the reference table in the Agency for Healthcare Research and Quality (AHRQ) guideline, after calculating the number of nurses for each hospital, the total minimum required sample size was 4,575 from 8 hospitals (Supplementary Table 1.1–1.2) (32). Inclusion criteria were nurses with at least 2 years of professional experience, at least 2 months of work in the current ward, and willingness to participate in the study. We excluded nurses who were not involved in direct patient care, those in administrative roles, and those on leave during the data collection period. A total of 7,170 (80.5%) nurses met the inclusion criteria and received a self-administered questionnaire. Among them, 5,003 nurses returned the completed survey, and after data cleaning, 4,982 valid responses were retained (69.5% valid response rate). We collected data from April to September 2025. Details of the study procedures have been described in our previous publication. The same sample and contemporaneously collected sociodemographic data were used, while this study focuses on nurses' patient safety knowledge and practices (30).

### 2.3 Instruments

To assess nurses' knowledge and practice related to patient safety culture and the associated influencing factors, the research team developed a Bengali version of the knowledge and practice on patient safety questionnaire. This self-administered questionnaire contains 10 knowledge and 10 practice items based on the National Patient Safety Strategic Plan of Bangladesh (27)**,** the Centers for Disease Control and Prevention (CDC), National Healthcare Safety Network (NHSN) Patient Safety Component Manual (2024) (34), the WHO Global Patient Safety Action Plan (2021–2030) (1), relevant literature, and expert input. The questionnaire was initially developed in English and then translated into Bengali using a forward-backwards translation approach to ensure linguistic and contextual appropriateness. Content validity was established through review by an expert panel consisting of a patient safety researcher, a clinical nursing expert, and additional subject specialists. Minor revisions were made based on their feedback. A pilot test was conducted among 20 nurses from a tertiary-level hospital outside the study setting to assess clarity, feasibility, and reliability. The 20-item knowledge and practices on patient safety questionnaire showed acceptable overall internal consistency (Cronbach's alpha = 0.745). The practice subscale had good reliability (Cronbach's alpha = 0.837), whereas the knowledge subscale had poor reliability (Cronbach's alpha = 0.382). Responses to knowledge questions on patient safety were scored as 0 for incorrect answers and 1 for correct answers. The total scores range from 0 to 10, with higher scores indicating better knowledge. The practice scale was a 5-point Likert scale scoring 1 = never, 2 = rare, 3 = sometimes, 4 = most of the time and 5 = always, and the total score ranged from 10 to 50. A higher score indicated better practice. In the questionnaire to assess PSC, we used a valid and reliable Hospital Survey on Patient Safety Culture 2.0 (HSOPSC) (30). We included socio-demographic questions on age, gender, nursing education, total professional experience, current hospital/ward tenure, and in-service training in nursing care, administration, and infection prevention and control.

### 2.4 Data collection procedures

After receiving written permission from the hospital authorities, we collected data in a structured manner. First, the principal investigator (PI) held a meeting in each hospital conference room with the ward in-charge and nurses from different wards. PI delivered a brief orientation about the purpose of the study, the questionnaire format, ethical considerations, inclusion/exclusion criteria, and the instructions for completion. Nurses who met the eligibility criteria and agreed to participate were provided with written informed consent, which was obtained by the PI and trained Research Assistants (RAs). Following consent, self-administered questionnaires were distributed in this session within the hospital setting. Subsequently, the PI and trained Research Assistants (RAs) visited each ward sequentially to distribute the remaining questionnaires and provide additional clarification whenever needed. Participants were instructed to complete the questionnaire independently, either immediately or within 2–3 weeks of distribution, after understanding the instructions in the hospital (one by one in each hospital). All completed questionnaires were collected anonymously, assigned unique identification numbers, and checked for completeness before data entry. The surveys contained no names or other personal identifiers and were returned through secure drop boxes placed in the duty and supervisor rooms. Trained RAs supervised the process and helped ensure the accuracy and quality of the data.

### 2.5 Ethical consideration

The study was carried out in accordance with the ethical principles outlined in the Declaration of Helsinki (35). Ethical clearance was granted by the National Research Ethics Committee of the Bangladesh Medical Research Council (Registration No.64002032025) prior to the commencement of the study.

### 2.6 Data analysis

The data were analyzed using SPSS for Windows version 31.0 (IBM Corp., Armonk, NY, USA). Listwise deletion was used to address 21 missing values. As the proportion of missing observations was low (<5%) and no evident systematic pattern of missing data was identified, a complete case analysis of 4,982 participants was performed. Knowledge items (*n* = 10) were dichotomized as correct=1 and incorrect=0, yielding a total knowledge score ranging from 0 to10. Practice items (*n* = 10) were measured using a 5-point Likert scale, with total scores ranging from 10 to 50. Negatively worded items were reverse-coded before analysis. Descriptive statistics are presented as mean and standard deviation (SD) for continuous knowledge and practice scores. As data were not normally distributed, the Mann–Whitney U and Kruskal–Wallis nonparametric tests were performed for bivariate comparisons. To account for potential clustering of participants within hospitals and estimate adjusted associations, linear mixed-effects models were conducted for knowledge and practice scores. Hospital was specified as a random-intercept effect, while relevant sociodemographic and professional characteristics were included as fixed effects. Models were estimated using maximum likelihood and adjusted regression coefficients (B) with 95% confidence intervals (CIs), and *p*-values were reported. Statistical significance was set at *p* < 0.05. Model assumptions were assessed through visual inspection of residual-versus-predicted-values scatterplots and normal Q–Q plots to evaluate homoscedasticity and residual normality. Multicollinearity among fixed-effect variables was evaluated using variance inflation factors (VIFs), with VIF values <10 indicating no substantial multicollinearity. The percentage of positive responses for each item and patient safety culture item and dimension were calculated according to the HSOPSC 2.0 user's guide and previously published studies (30). Dimensions with a positive response rate (PRR) ≥ 75% were categorized as areas of strength, 50%–74% as areas with potential for improvement, and <50% as areas of weakness (32, 35). Working wards were categorized into general wards (medicine, surgery, orthopedics, pediatrics/neonatology) and specialized wards (emergency, ICU, cardiology/CCU, operating theater, urology/nephrology, oncology/hematology, neurology/neurosurgery).

## 3 Results

### 3.1 Sociodemographic characteristics of the participants

A total of 4,982 nurses participated in the study. The mean age was 37.1 years (SD ± 7.8), with most aged 31–40 years (52.2%), and the majority were female (91.1%). Most held a diploma (59.8%), with a mean professional experience of 12.2 ± 7.1 years. Nurses were mainly from medicine (23.1%), surgery (13.8%), and pediatric/neonatal wards (11.6%). More than half (52.7%) had no in-service training, and training in IPC/patient safety (3.4%) and ICT/event reporting (2.4%) was notably limited (Table 1). Compared to the overall sample, DMCH had higher proportions (52.4%) of BSc/master's nurses and higher in-service training coverage in KMCH and MMCH (53.8% and 52.2%), while RMCH and KMCH were dominated by nurses with diploma education (73.2% and 72.8%), and RpMCH had less training coverage. SMCH had the youngest workforce with relatively better IPC/patient safety training exposure, while KMCH had the oldest (39.0 ± 8.0**)** workforce (not shown in the Table).

**Table 1:** Sociodemographic characteristics and their association with patient safety knowledge and practice (*n* = 4,982).

Variables	Categories	Frequency (%)	Knowledge			Practice		
			Mean	SD	*p*-value	Mean	SD	*p*-value
Age (years)^a^(Mean ±SD = 37.1 ± 7.8)	≤ 30	1,070 (21.5)	5.42	1.87	0.759	29.93	8.36	<0.001
	31- 40	2,602 (52.2)	5.43	1.82		28.85	7.62	
	> 40	1,310 (26.3)	5.47	1.77		28.22	7.43	
Gender^b^	Male	441 (8.9)	5.27	1.83	0.039	29.39	7.21	0.059
	Female	4,541 (91.1)	5.46	1.82		28.87	7.81	
Nursing education^a^	Diploma in nursing (3 years of education)	2,977 (59.8)	5.34	1.84	<0.001	28.78	7.71	0.039
	BSc in nursing (4 years of education)	1,299 (26.1)	5.58	1.79		29.02	7.90	
	Master and above	706 (14.2)	5.63	1.74		29.31	7.69	
Professional experience (years) (Mean ±SD = 12.2 ± 7.1)	≤10	2,757 (55.3)	5.39	1.84	0.043	28.98	7.82	0.430
	> 10	2,225 (44.7)	5.50	1.79		28.83	7.68	
Hospital experience (years)^a^ (Mean ±SD = 8.6 ± 6.1)	< 5	1,884 (37.8)	5.42	1.84	0.855	29.35	8.06	0.003
	6–11	1,984 (39.8)	5.45	1.82		28.83	7.58	
	≥ 12	1,114 (22.4)	5.46	1.77		28.33	7.52	
Working ward^a^	Medicine	1,149 (23.1)	5.24	1.82	<0.001	28.84	7.64	<0.001
	Surgery	690 (13.8)	5.59	1.73		28.73	8.23	
	Orthopedics/orthopedic surgery	218 (4.4)	5.37	1.83		30.26	7.61	
	Gynecology and obstetrics	428 (8.6)	5.54	1.85		27.75	7.25	
	Pediatric/ neonatology	576 (11.6)	5.78	1.87		28.43	8.08	
	Emergency	215 (4.3)	5.01	1.80		31.53	8.60	
	Intensive care unit	449 (9.0)	5.48	1.76		29.71	6.85	
	Cardiology/CCU	263 (5.3)	5.03	2.13		27.49	5.72	
	Operation theater	392 (7.9)	5.58	1.78		27.23	8.05	
	Urology/nephrology	206 (4.1)	5.10	1.61		29.43	7.35	
	Oncology/hematology	189 (3.8)	5.45	1.68		31.34	8.26	
	Neurology/neurosurgery	207 (4.2)	5.91	1.62		30.15	7.89	
Experience in ward years^a^ (Mean ±SD = 3.9 ± 3.2)	< 2 years	2,097 (42.1)	5.43	1.81	0.489	29.14	7.97	0.063
	3–5 years	1,240 (24.9)	5.49	1.83		29.00	7.71	
	≥ 6 years	1,645 (33.0)	5.41	1.82		28.57	7.50	
Have in-service training^b^	Yes	2,356 (47.3)	5.58	1.80	<0.001	28.63	7.74	0.019
	No	2,626 (52.7)	5.32	1.82		29.18	7.76	
Type of in-service training^a^	Nursing administration and management	455 (9.1)	5.39	1.84	0.081	28.55	7.93	0.033
	ICT and event reporting	119 (2.4)	5.34	1.87		27.20	7.50	
	IPC and patient safety	167 (3.4)	5.72	1.78		29.64	7.35	
	Patient care services	1,144 (23.0)	5.63	1.85		28.68	7.88	
	Other	471 (9.5)	5.65	1.63		28.58	7.38	
Types of other training^b^	Orientation	346 (7.4)	5.63	1.57	0.286	28.15	7.67	0.011
	ICU	125 (2.5)	5.71	1.80		29.77	6.39	

a Kruskal-Walli's test.

b Mann–Whitney U.

IPC, Infection Prevention and Control; ICT, Information and Communication Technology; ICU, Intensive Care Unit.

General ward: (mean knowledge and practice 5.50 and 28.80); Specialized ward: (mean knowledge and practice 5.37 and 29.55).

### 3.2 Level of knowledge on patient safety among the participants

The overall mean knowledge score was 5.44 (SD ±1.82). Among the eight hospitals, the highest knowledge mean score was observed in DMCH (5.96, SD ±1.71), followed by SMCH (5.67, SD ±2.00). In contrast, lower mean scores were found in CMCH (4.94, SD ±1.78) and SBMCH (4.98, SD ±1.87) (Supplementary Table 2). At the item level of knowledge on patient safety, the highest correct responses were for knowing the hospital incident reporting system (Item 1, 73.8%), the color code for infectious waste disposal (Item 7, 72.1%), and the rights of medication administration (Item 8, 65.4%). The lowest scores were for the reporting method used for patient safety adverse events (Item 2, 9.8%), correct disposal of needles (Item 10, 40.9%), and identifying which events should be reported (49.3%), respectively (Table 2). Across the eight hospitals, item-specific knowledge frequently diverged from overall benchmarks. For instance, knowledge of hospital incident reporting systems ranged from 65.8% (SBMCH) to 84.4% (KMCH), while knowledge of hospital patient safety teams varied from 47.6% (CMCH) to 83.4% (DMCH). Knowledge regarding appropriate reporting methods remained critically low, spanning from 3.4% (KMCH) to 13.3% (DMCH). Similarly, the ability to correctly identify reportable adverse events fluctuated between 34.2% (SBMCH) and 61.3% (DMCH), respectively (Supplementary Table 2).

**Table 2:** Distribution of correct responses on patient safety knowledge items among the nurses (*n* = 4,982).

Knowledge of patient safety items	%
1. Do you know about your hospital incident (adverse events) reporting system?	73.8
2. Which correct reporting method is used for reporting adverse events in your hospital?	9.8
3. Do you know about the “National Hospital Policy and Procedure” for adverse event/incident reporting system?	53.2
4. Do you know the ‘Hospital Patient Safety Quality Improvement Team’ exists in your hospital?	63.7
5. Which should be reported as an adverse event/incident?	49.3
6. Recommended hand hygiene times according to the WHO, EXCEPT	60
7. What is the appropriate color code for disposing of infectious waste?	72.1
8. All are Rights of Medication Administration, EXCEPT	65.4
9. Which is NOT considered an adverse event in a hospital?	55.8
10. What is the correct method for disposing of needles after use?	40.9
Mean ± SD (range: 0–10)	5.44 ± 1.82

### 3.3 Level of practice on patient safety among the participants

The overall participants' mean score was 28.92 (SD 7.76) for patient safety practice. Among 10 practice items, 48.6% reported never using the incident reporting form, while only 2.4% reported frequent use. The use of standardized communication methods such as Situation, Background, Assessment, and Recommendation (SBAR) was also limited, with 26.4% never and 28.4% rarely used. Practices such as patient identification 44.0% and communication during shift changes 41.5% were reported as being performed most of the time or always. However, medication safety practices, including double-checking medications 34.0% and verifying allergy history 39.3%, were most reported in the “sometimes” category. Similarly, adherence to standard precautions 34.4%, use of checklists during high-risk procedures 32.2%, reporting of medication errors 32.4%, and reporting of patient falls 34.4% were reported in the “sometimes” category (Table 3).

**Table 3:** Distribution of patient safety practice items among nurses (*n* = 4,982) presented as percentage (%).

Practice of patient safety items	Never	Rarely	Sometimes	Most of the time	Always	Mean (SD) (range:10–50)
	%	%	%	%	%	
1. Do you use the Incident Reporting Form (developed by the Quality Improvement Secretariat-QIS) to report adverse events/incidents?	48.6	28.8	15.7	4.6	2.4	28.92 (7.76)
2. Do you verify patient identity using name, date of birth, and hospital registration number?	6.0	28.5	21.5	23.6	20.4	
3. Do you double-check medication labels and doses with your colleague before administration?	9.3	23.6	34.0	18.4	14.7	
4. Do you practice standard precautions, such as handwashing before and after any procedure?	5.6	27.4	34.4	21.2	11.4	
5. Do you use a standardized communication method like SBAR to report patient condition to physicians?	26.4	28.4	26.1	13.7	5.4	
6. Do you clearly and concisely share critical patient information during shift changes?	5.3	22.3	30.8	26.0	15.5	
7. Do you use a standard checklist while performing high-risk procedures?	8.0	23.5	32.2	23.5	12.9	
8. Do you report medication errors if it occurs?	6.5	27.0	32.4	22.3	11.7	
9. Do you check a patient's allergy history before administering medications?	6.9	25.4	39.3	21.9	6.5	
10. If a patient falls from bed, do you report it as an accident?	9.2	26.5	35.4	18.4	10.5	

### 3.4 Differences in patient safety knowledge and practice score by sociodemographic characteristics

Patient safety knowledge scores were significantly associated with gender (*p* *=* 0.039), nursing education level (*p* < 0.001), professional nursing experience (*p* *=* 0.043), working ward (*p* < 0.001) and receipt of in-service training (*p* < 0.001). Knowledge score was higher among female nurses than male nurses (mean, 5.46 vs. 5.27), and increased with higher nursing educational level, from 5.34 for diploma to 5.63 among those with master's or higher education. Nurses with >10 years of professional experience had higher knowledge than ≤ 10 years. Nurses who had received in-service training showed higher scores than those who had not (5.58 vs. 5.32).

Patient safety practice scores were significantly associated with age (*p* < 0.001), nursing education level (*p* *=* *0.039*), hospital experience (*p* *=* *0.003*), working ward (*p* < 0.001), receipt of in-service training (*p* *=* *0.019*), types of in-service training (*p* *=* *0.033*) and other training (*p* *=* *0.01)*. Practice scores were highest among young nurses aged ≤ 30 years (29.93) and declined across older age groups. Nurses with higher educational attainment showed higher practice scores, increasing from 28.78 among diploma holders to 29.31 among those who have master's and above. Nurses who had received in-service training had lower practice scores than those who had not (28.63 vs. 29.18) (Table 1).

### 3.5 Patient safety culture across eight hospitals (*n* = 4,982)

The eight hospitals showed an overall moderate level of patient safety culture with a positive response rate of 56% (PRR 50%–74%) as potential for improvement (moderate patient safety culture), which was reported in our previous paper (30). Across hospitals, the overall PRR ranged from 50% to 65%, with the highest observed in KMCH (65%) and DMCH (63%), and lower values in RpMCH (50%) and SBMCH (51%). Variation across hospitals was also noted for individual dimensions, particularly in communication about errors (53%–92%) and reporting patient safety events (4%–42%), respectively (Supplementary Table 4).

### 3.6 Patient safety events reporting and rating among the participants (*n* = 4,982) and across 8 hospitals

#### 3.6.1 Patient safety events reporting

Figure 2 shows that overall, 28.4% of nurses reported no patient safety events in the past 12 months, while 43.7% reported 1–2 events, followed by 18.7% reporting 3–5 events. Smaller proportions reported 6–10 events (5.6%) and ≥11 events (3.7%). Across hospitals, the proportion reporting no events was highest in KMCH (39.1%), SMCH (36.3%), and RpMCH (34.9%), and lowest in MMCH (19.8%) and DMCH (21.4%) (Supplementary Table 5).

**Figure 2:**
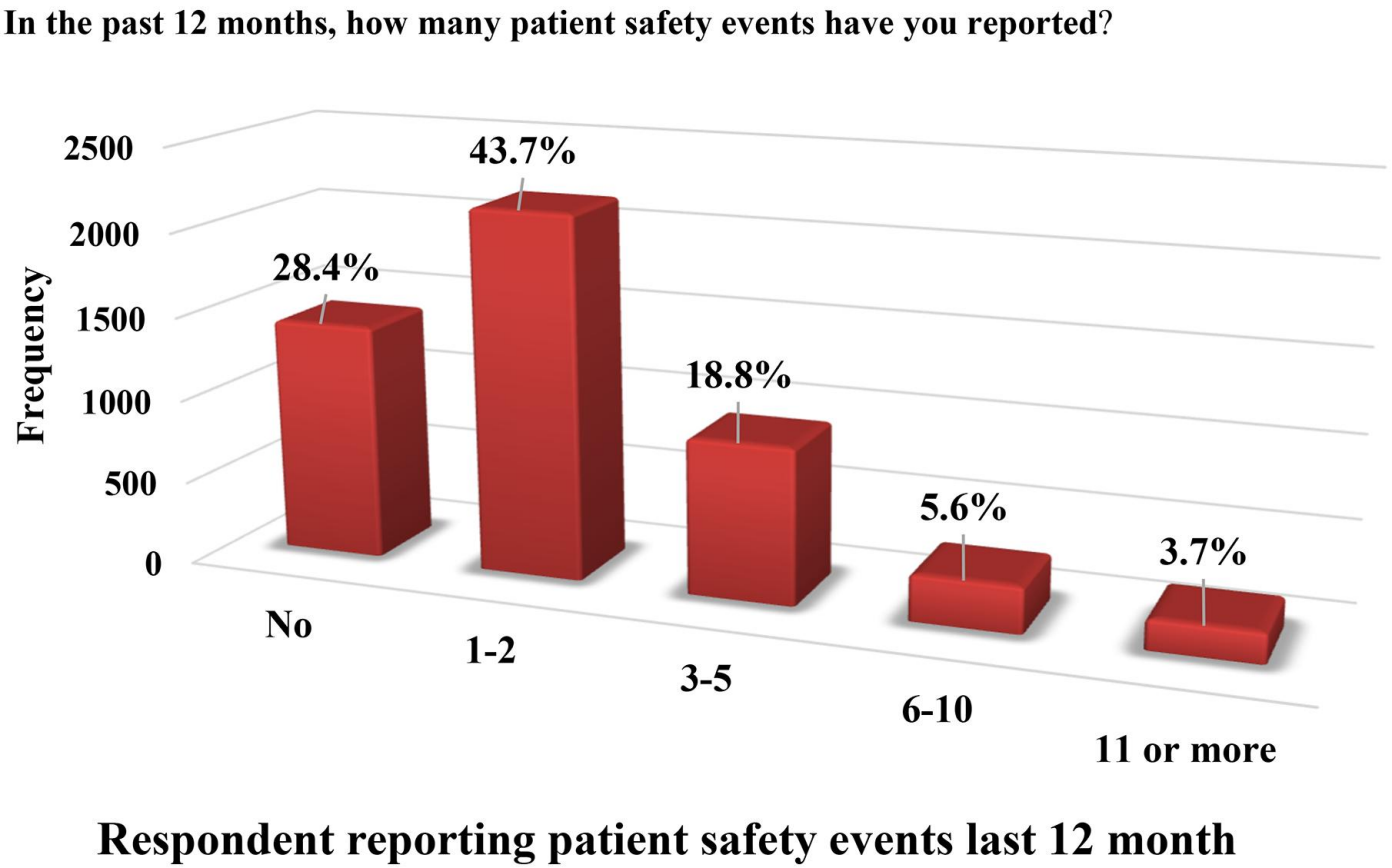
Frequency of patient safety adverse events reported among the participants.

#### 3.6.2 Patient safety adverse events reporting by the participants (*n* = 4,982)

##### 3.6.2.1 Patient safety rating

Regarding patient safety rating, 46.5% of participants rated their hospitals as “good,” followed by “fair” (29.8%), “very good” (14.3%), and “excellent” (6.0%), while 3.4% rated safety as “poor” (Figure 3).

**Figure 3:**
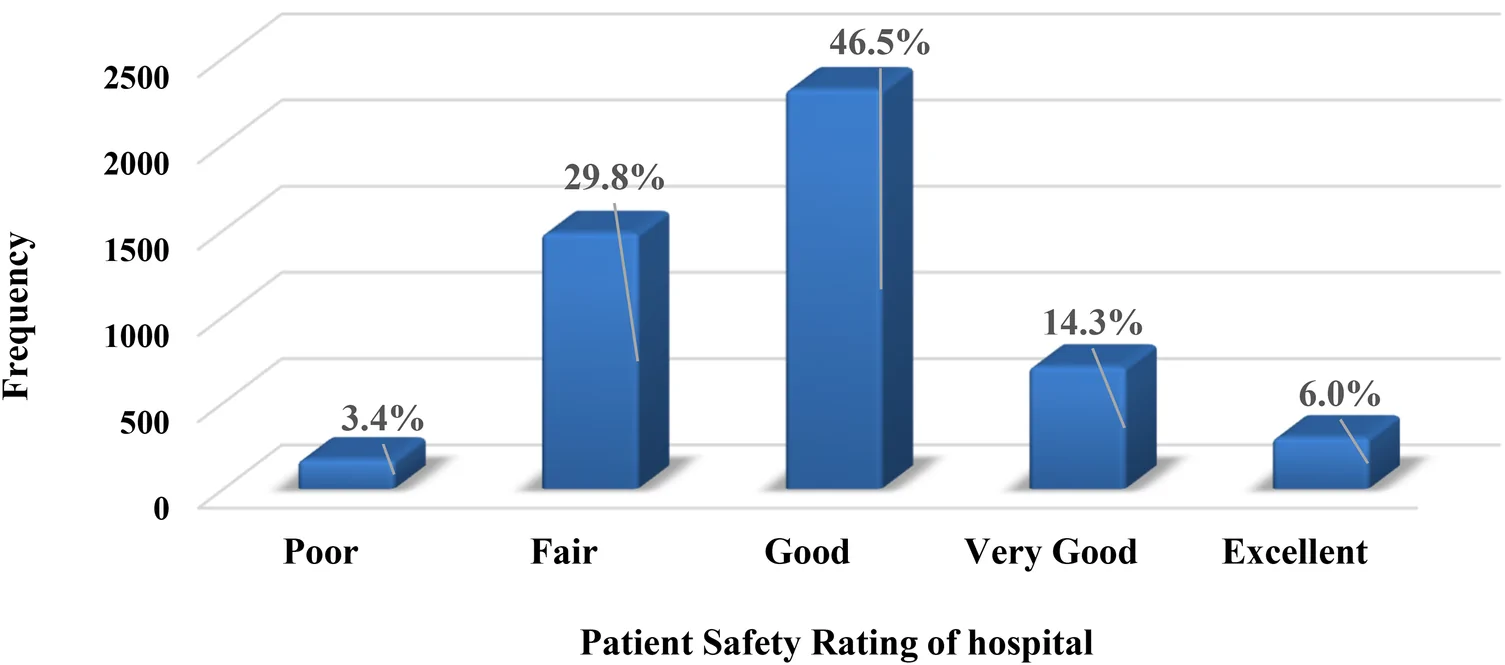
Frequency of patient safety ratings of working hospitals among the participants.

Across hospitals, higher proportions of “excellent” ratings were observed in KMCH (21.5%) and DMCH (9.9%), whereas a higher proportion of “poor” ratings was reported in SBMCH (11.4%) (Supplementary Table 5).

##### 3.6.2.2 Factors associated with patient-safety knowledge and practices: linear mixed-effects model

Diagnostic assessment of the linear mixed-effects models revealed no substantial evidence of heteroscedasticity based on residual-versus-fitted plots. Normal Q–Q plots indicated that residuals were approximately normally distributed, although minor deviations were observed at the distribution tails. Multicollinearity diagnostics indicated no substantial collinearity among the predictors, with VIF values ranging from 1 to 5.9. Overall, no serious violations of model assumptions were identified. Professional experience, gender, education, working ward, and in-service training were significantly associated with patient-safety knowledge scores. Nurses' professional experience (B = 0.019, 95% CI: 0.003–0.034, *p* = 0.018) and in-service training (B = 0.346, 95% CI: 0.168–0.523, *p* < 0.001) were positively associated with knowledge. However, gender (male) (B = −0.181, 95% CI: −0.360 to −0.001, *p* = 0.049) and diploma in nursing education (B = −0.168, 95% CI: −0.316 to −0.020, *p* = 0.027) were negatively associated with knowledge. For working ward, medicine, surgery, Orthopedics/orthopedic surgery, gynecology and obstetrics, pediatrics/neonatology, intensive care, cardiology/CCU, urology/nephrology, and oncology/hematology (all, *p* < 0.05) were negatively associated with knowledge. Nursing administration and management training was also negatively associated with knowledge scores (B = −0.475, 95% CI: −0.711 to −0.240, *p* < 0.001). The hospital-level ICC was 0.048, indicating that approximately 4.8% of the unexplained variance in knowledge scores was attributable to differences between hospitals (Table 4).

**Table 4:** Factors associated with nurses’ patient-safety knowledge using a linear mixed-effects model.

a. Factor	Coefficient (B)	95% CI lower	95% CI upper	*p*-value
Age (years)	−0.011	−0.025	0.003	0.128
Professional experience (years)	0.019	0.003	0.034	0.018
Experience in current hospital (years)	−0.004	−0.018	0.011	0.613
Experience in current ward (years)	−0.006	−0.022	0.011	0.491
Gender				
Male (ref: female)	−0.181	−0.360	−0.001	0.049
Education				
Diploma in nursing (ref: master and above)	−0.168	−0.316	−0.020	0.027
BSc in nursing (ref: master and above)	−0.015	−0.178	0.148	0.856
Work ward				
Medicine (ref: neurology/neurosurgery)	−0.646	−0.908	−0.384	<0.001
Surgery (ref: neurology/neurosurgery)	−0.359	−0.634	−0.084	0.010
Orthopedics/orthopedic surgery (ref: neurology/neurosurgery)	−0.967	−1.307	−0.627	<0.001
Gynecology and obstetrics (ref: neurology/neurosurgery)	−0.498	−0.792	−0.203	0.001
Pediatric/ neonatology (ref: neurology/neurosurgery)	−0.878	−1.202	−0.553	<0.001
Emergency (ref: neurology/neurosurgery)	−0.282	−0.580	0.016	0.063
Intensive care unit (reference: neurology/neurosurgery)	−0.362	−0.698	−0.026	0.035
Cardiology/CCU (ref: neurology/neurosurgery)	−0.349	−0.642	−0.055	0.020
Operation theater (ref: neurology/neurosurgery)	−0.111	−0.394	0.171	0.440
Urology/nephrology (ref: neurology/neurosurgery)	−0.768	−1.109	−0.428	<0.001
Oncology/hematology (ref: neurology/neurosurgery)	−0.604	−0.952	−0.256	0.001
In-service training				
Have Training (ref: no)	0.346	0.168	0.523	<0.001
Type of service training				
Nursing administration and management (ref: others)	−0.475	−0.711	−0.240	<0.001
ICT and event reporting (ref: others)	−0.314	−0.669	0.042	0.084
IPC and patient safety (ref: others)	0.018	−0.295	0.331	0.908
Patient care services (ref: others)	−0.098	−0.293	0.097	0.326
Intercept	6.120	5.574	6.666	<0.001

b. Random-effects parameters	
Parameter	Estimate
Hospital-level random-intercept variance	0.155
Residual variance	3.067
Intraclass correlation coefficient (ICC)	0.048

B represents the change in knowledge score associated with each predictor relative to the specified reference category or per one-unit increase for continuous variables; CI = confidence interval; ICC = intraclass correlation coefficient. Statistically significant at <0.05.

Linear mixed-effects model with a random intercept for hospital (8 hospital clusters), maximum likelihood (ML), *n* = 4,982. The dependent variable was the total patient-safety knowledge score (range: 0–10).

Nurses' age, professional experience, work ward, and in-service training were significantly associated with nurses' patient-safety practice scores. Professional experience was positively associated with patient safety practice (B = 0.167, 95% CI: 0.100–0.234, *p* < 0.001). Participants' age (B = −0.181, 95% CI: −0.242 to −0.121, *p* < 0.001) and in-service training (B = −0.915, 95% CI: −1.676 to −0.153, *p* < 0.019) were negatively associated with practices. For working ward, medicine, surgery, pediatrics/neonatology, emergency and operation theater (all, *p* < 0.05) were negatively associated with patient safety practices. The hospital-level ICC was 0.053, suggesting that approximately 5.3% of the unexplained variance in practice scores can be attributed to differences among hospitals (Table 5).

**Table 5:** Factors associated with nurses’ patient-safety practice scores using a linear mixed-effects model.

a. Factors	Coefficient (B)	95% CI lower	95% CI upper	*p*-value
Age (years)	−0.181	−0.242	−0.121	<0.001
Professional experience (years)	0.167	0.100	0.234	<0.001
Experience in current hospital (years)	0.007	−0.055	0.069	0.819
Experience in current ward (years)	0.040	−0.030	0.110	0.263
Gender				
Male (ref: female)	−0.507	−1.280	0.265	0.198
Education				
Diploma in nursing (ref: master and above)	−0.072	−0.710	0.565	0.824
BSc in nursing (ref: master and above)	−0.133	−0.835	0.570	0.712
Work ward				
Medicine (ref: neurology/neurosurgery)	−1.150	−2.276	−0.024	0.045
Surgery (ref: neurology/neurosurgery)	−1.306	−2.487	−0.125	0.030
Orthopedics/orthopedic surgery (ref: neurology/neurosurgery)	0.910	−0.553	2.373	0.223
Gynecology and obstetrics (ref: neurology/neurosurgery)	−0.711	−1.977	0.554	0.271
Pediatric/ neonatology (ref: neurology/neurosurgery)	−2.996	−4.392	−1.601	<0.001
Emergency (ref: neurology/neurosurgery)	−3.050	−4.332	−1.768	<0.001
Intensive care unit (reference: neurology/neurosurgery)	0.118	−1.327	1.563	0.873
Cardiology/CCU (ref: neurology/neurosurgery)	−2.094	−3.356	−0.832	0.001
Operation theater (ref: neurology/neurosurgery)	−1.902	−3.118	−0.686	0.002
Urology/nephrology (ref: neurology/neurosurgery)	−0.803	−2.268	0.661	0.282
Oncology/hematology (ref: neurology/neurosurgery)	0.960	−0.535	2.456	0.208
In-service training				
Have Training (ref: no)	−0.915	−1.676	−0.153	0.019
Type of service training				
Nursing administration and management (ref: others)	0.368	−0.644	1.381	0.476
ICT and event reporting (ref: others)	−1.077	−2.606	0.451	0.167
IPC and patient safety (ref: others)	0.984	−0.362	2.330	0.152
Patient care services (ref: others)	0.584	−0.254	1.422	0.172
Intercept	34.882	32.498	37.266	<0.001

b. Random-effects parameters	
Parameter	Estimate
Hospital-level random-intercept variance	3.154
Residual variance	56.725
Intraclass correlation coefficient (ICC)	0.053

B represents the change in knowledge score associated with each predictor relative to the specified reference category or per one-unit increase for continuous variables; CI = confidence interval; ICC = intraclass correlation coefficient.

Statistically significant at <0.05.

Linear mixed-effects model with a random intercept for hospital (8 hospital clusters), maximum likelihood (ML),.

*n* = 4,982. The dependent variable was the total patient-safety practices score (range: 10–50).

## 4 Discussion

To the best of our knowledge, this is the first multicenter study to explore nurses' patient safety knowledge, practices and associated sociodemographic and professional factors in tertiary-level hospitals in Bangladesh. The findings identified variability in participants' knowledge and practices across the assessed items. Overall patient safety ratings by the nurses were good with a moderate level of PSC across hospitals; however, adverse event reporting was inadequate (no reporting to 1–2 event reporting 72.1%). Participants' higher educational levels, working ward, and in-service training were associated with patient safety knowledge, while younger age and working ward were associated with practice. In the mixed-effects model, nursing education, professional work experience, working ward, and type of in-service training were associated with knowledge scores, whereas age and working ward were associated with practice scores.

### 4.1 Nurses' knowledge of patient safety

The study revealed that, across the eight hospitals, correct-response rates for patient safety knowledge varied substantially, with several items showing notably low response rates. In Bangladesh, the “National Hospital Policy and Procedure’ provides a framework for hospital patient safety, and each hospital should have its own “Patient Safety Quality Improvement Team (QIT)’ for monitoring adverse events (27, 36). In this study, nurses' correct-response rates were 53.2% for knowledge of the National Hospital Policy and Procedure on events reporting and 63.7% for knowledge of their hospital's Patient Safety Quality Improvement Team.

Although 73.8% of nurses reported awareness of the hospital incident reporting system, only 9.8% correctly identified the appropriate method for reporting adverse events, suggesting limited familiarity with standardized reporting procedures.

In Bangladesh, the Quality Improvement Secretariat, Ministry of Health and Family Welfare (27) developed a standardized Incident Report Form (IRF) for documenting incidents, injuries and other patient-related events for hospitals. However, in this study, nurses reported conventional registered books more frequently than the standardized IRF for reporting incidents/ adverse events, while some also reported incidents verbally to their supervisors or relevant authorities. These findings suggest limited familiarity with the standardized IRF and highlight the need for hospital patient-safety Quality Improvement Teams (QITs) to strengthen awareness, availability, and implementation of the standardized IRF-based reporting system.

Nurses are expected to recognize patient-safety adverse events and report them within the stipulated timeframe. However, only 49.3% correctly identified an event that should be reported, while 55.8% correctly recognized an event that should not be considered an adverse event. These findings suggest gaps in nurses' ability to distinguish between reportable and non-reportable patient-safety events. A hospital-based study conducted in Nepal similarly reported that the majority of the respondent nurses had poor knowledge of patient safety incident reporting (73.1%) (37). Evidence from South African and other African studies also highlights knowledge gaps and weak leadership as key barriers to effective patient safety event reporting (38, 39).

Regarding knowledge of clinical safety practices, the proportion of correct responses varied from 40% to 72%. Knowledge of the proper disposal of needles was particularly low, highlighting an important gap in occupational safety knowledge. This gap may have implications for safe sharps handling and the prevention of needlestick injuries and potential exposure to bloodborne pathogens among them. This finding is consistent with previous studies, where it was observed that improper needle disposal significantly increases the risk of needlestick injuries and facilitates the spread of bloodborne communicable diseases, including hepatitis, among nurses (40, 41). To address this knowledge gap, used needles should be disposed of promptly in puncture-resistant sharps containers in the working ward. Hospital Quality Improvement Teams (QITs) should consider targeted education and training on safe sharps disposal, together with ensuring the availability of appropriate healthcare waste-disposal resources. Comparable findings have been reported among ICU nurses in Egypt, where more than half demonstrated poor knowledge of clinical patient safety practices, which can be improved through an in-service training program (42, 43).

### 4.2 Nurses' practices on patient safety

The study revealed that, across eight hospitals the proportion of correct responses varied substantially across items with several patient safety practice items showing lower response rates. Nurses’ reported patient safety practices varied across eight hospitals. In particular, for patient-safety event reporting, a substantial proportion of nurses reported performing these practices only “sometimes” or “never.” Specifically, 93.1% reported using the incident reporting form, 65.9% reported medication errors, and 71.1% reported patient falls at these frequencies. These findings suggest that patient-safety event reporting practices were not consistently performed among the participating nurses. Such poor reporting practices are highly alarming because incident reporting is essential for identifying risk, preventing recurrence, and improving hospital patient safety (44).

In this study, 71.6% of nurses reported at least one patient safety event in the previous year, whereas 28.4% reported no events. Despite the occurrence of patient-safety events, formal incident-reporting practices were not consistently reported. This discrepancy may indicate potential barriers to incident reporting; however, specific barriers, such as unfamiliarity with reporting forms, reliance on register books, fear of blame, or the absence of a structured reporting system, were not directly assessed in this study. Future research should explore the barriers and facilitators influencing patient-safety incident reporting in tertiary-level hospitals in Bangladesh. In addition, examining the timeliness and adequacy of follow-up after incident reporting may help determine whether institutional responses influence nurses' engagement with and confidence in patient-safety reporting systems. Previous research has identified weak leadership, ineffective reporting systems e.g., limited availability of reporting forms, and inadequate feedback regarding reported incidents have been identified as potential barriers to the implementation of patient safety practices and safe clinical practice (45). Future intervention studies should evaluate strategies to strengthen leadership commitment, ensure accessible incident-reporting tools, and establish a non-punitive feedback system to improve incident reporting and patient safety performance in tertiary hospitals in Bangladesh.

Regarding patient-safety communication practices, the use of SBAR as a standardized communication tool (46) was infrequent, with 80.9% of nurses reported using it from ’Sometimes' to “Never”. This finding suggests limited use of standardized communication practices among the participating nurses and highlights an area for strengthening structured communication in patient care. The study findings highlight ongoing communication breakdowns that remain common in clinical practices and underscore the need to promote SBAR use to strengthen handoff processes and patient safety, as it is a recommended structured communication tool (47). In addition, limited sharing of patient information during shift changes was observed as 58.4% of nurses reported this practice only “Sometimes” to “Never,” which may increase the risk of errors and compromise patient safety (48).

Nurses reported clinical safety practices, including patient identity verification, double-checking medication labels, adherence to standard precautions for hand hygiene, use of a checklist for high-risk procedures, and assessment of patients' allergic history before medication administration, were reported as “sometimes,” “rarely,” or “never” by 56.0%–71.6% of nurses. This pattern indicates that these safety practices were not consistently performed and highlights areas requiring further attention to strengthening routine patient-safety practices. Previous studies have shown that verifying patient identity by using multiple identifiers and cross-checking of medications by peer nurses before administration can help to prevent medication errors (49, 50).

Nurses reported inconsistent hand-hygiene practices and limited use of safety checklists for high-risk procedures, including blood transfusion among nurses. These self-reported practice gaps highlight areas requiring attention to strengthen adherence to standard precautions and the consistent use of safety checklists in routine clinical practice. Prior research has shown that improved hand hygiene compliance among nurses is associated with better patient outcomes and fewer healthcare-associated infections (51). These findings may also be influenced by broader system-related factors; however, such factors were not measured in this study. Previous studies have reported that staffing shortages and workload pressure may adversely affect patient safety practices and incident reporting (52, 53). Moreover, previous research suggests that competing clinical demands and the absence of a structured incident reporting system may further hinder incident reporting and patient safety practices among the nurses (27, 54).

### 4.3 Association of knowledge and practice with sociodemographic characteristics

This study identified significant associations between nurses' patient safety knowledge and practice scores and several sociodemographic characteristics, including age, gender, educational level, experience, professional experience, working ward, and in-service training. Professional experience and in-service training were positively associated with knowledge scores, whereas gender, educational level, and work unit showed significant associations with knowledge in the adjusted linear mixed-effects model. Practice scores were significantly associated with age, professional experience, work unit, and in-service training.

Nurses with higher educational attainment had higher patient-safety knowledge scores. In the adjusted linear mixed-effects model, nurses in the lower educational categories had significantly lower knowledge scores compared with those with a master's degree or higher. Female nurses also had higher knowledge scores than male nurses, with male gender showing a significant negative association with knowledge scores compared with female nurses. These findings are consistent with previous research suggesting that higher educational attainment may be associated with better patient-safety knowledge among nurses (55). The observed gender difference may partly reflect the predominance of women in the nursing workforce in Bangladesh (23). However, the underlying reasons for this association were not directly assessed in the present study.

Nurses working in special wards and those without in-service training had lower knowledge of patient safety knowledge scores, which may reflect differences in access to continuing education and fewer structured learning opportunities in Bangladesh. These findings are relevant because previous studies have reported that training may improve nurses' adverse-event reporting practice (56). The observed association highlights the need to further evaluate the content, accessibility, and effectiveness of regular training on patient safety, infection prevention and control (IPC), and adverse-event reporting. Future studies should examine whether training needs differ by ward type, professional experience, and clinical role.

In terms of practice, younger nurses had higher patient safety practice scores. This association may reflect differences in exposure to contemporary patient-safety education, updated clinical protocols, and evidence-based practice. Younger nurses may also have had more recent exposure to formal nursing education and current clinical guidelines. However, these potential explanations were not directly assessed in the present study. Prior studies' findings showed higher educational attainment may further enhance critical thinking and the ability to translate safety knowledge into clinical practice (57, 58) and better awareness of quality and safety issues among this group.

Unexpectedly, more experienced nurses and those who had received in-service training had lower practice scores (negatively associated with practices). This finding may suggest that training exposure alone may not necessarily translate into consistent patient-safety practices. The content, recency, relevance, and opportunities for applying training in clinical settings may influence its potential contribution to practice and warrant further investigation. However, evidence suggests that targeted and practical patient-safety training can improve nurses' safety knowledge and practices (57, 59).

The reasons for the lower practice scores observed among these nurses cannot be determined from the present data. Previous studies suggest that workload pressure, clinical supervision, reinforcement of safety protocols, and post-training monitoring may influence nurses' patient-safety practices (56); however, these factors were not measured in this study. Similarly, lower practice scores among nurses working in general wards may reflect contextual factors such as high patient workloads, staffing constraints, and competing clinical and support activities, which may limit opportunities for consistent implementation of patient-safety practices (60). We conducted the study across eight hospitals and observed a low clustering effect for knowledge and practice, indicating that the findings primarily reflect individual-level variation rather than hospital-level differences.

### 4.4 Nurses' patient safety culture and rating

Nurses across eight tertiary hospitals demonstrated a moderate patient safety culture (overall PRR 50%–65%). KMCH recorded the highest score, whereas RpMCH and SBMCH showed the lowest. Although all hospitals had similar institutional status, these differences likely reflect variation in leadership commitment, resource allocation**,** and staff training, which appeared stronger in KMCH and DMCH. Overall patient safety culture in eight hospitals may also be influenced by a blame-oriented culture, heavy workload, and lack of reporting systems, along with regional variations in staffing shortages (61, 62). Patient safety ratings were 33.2% from “Poor” to “Fair” compared to 66.8% from “Good” to “Excellent”. In Bangladesh, comprehensive patient safety incident event reporting systems remain limited, mirroring LMICs and the South Asian setting, where underreporting is often driven by blame-oriented culture, workload pressure, and weak reporting systems (63, 64).

### 4.5 Strengths and limitations

This multicenter study conducted across eight tertiary-level medical college hospitals in Bangladesh with a large sample provides descriptive evidence and moderate insights on nurses’ patient safety knowledge and practices. The findings represent a broader view for nurses working at tertiary hospital in to nurses in Bangladesh and may also have relevance for other LMIC contexts. However, self-reported data, convenience sampling, and the selection of one government hospital per administrative division may have introduced social desirability bias and limited national representativeness. The cross-sectional design also restricts causal inference and assessment of temporal changes. In addition, the findings primarily generalize to nurses working in tertiary-level hospitals and do not capture the perspectives of other healthcare professionals or those in primary- and secondary-level hospitals in Bangladesh. Furthermore, the knowledge subscale has low reliability, the scale assessed distinct areas of patient safety knowledge, exhibiting heterogeneous responses. We used a highly homogeneous population (nurses) with similar educational backgrounds, which may reduce true score variance and contribute to low reliability, resulting in lower-than-expected internal consistency among the question items. Additionally, the knowledge subscale requires further refinement and validation, including assessment of test–retest reliability and item performance among healthcare professionals from diverse professional backgrounds and hospital tiers before its future use.

## 5 Conclusion

Nurses in tertiary-level hospitals in Bangladesh demonstrated average knowledge and patient-safety practices. Although the overall patient safety culture was moderate across hospitals with adverse event reporting remained notably low. Professional experience was positively associated with knowledge and practice. Nurses with in-service training were positively associated with knowledge. In contrast, male gender compared to female and diploma nurses compared to master's and above were negatively associated with lower knowledge. Furthermore, more experienced senior nurses and those who had not received in-service training demonstrated poorer patient safety practices. Therefore, targeted and context-specific in-service training programs focusing on patient safety, infection prevention and control (IPC), and adverse event reporting are essential to improve nurses' knowledge and practices. QIT should take a leading role in strengthening the monitoring and functionality of adverse event reporting systems to enhance patient safety practices. Additionally, hospital authorities need to ensure the consistent availability of essential logistics and supplies to support effective hand hygiene, waste management, and adverse event reporting among nurses. At the national level, regulatory bodies such as the Directorate General of Nursing and Midwifery (DGNM) and the Directorate General of Health Services (DGHS) should implement robust monitoring and evaluation strategies to improve nurses' knowledge and practices, ultimately strengthening the quality of care and patient safety in Bangladesh. Future studies should include all graduated nurses, who may be particularly vulnerable to errors and require targeted patient-safety education and safety-culture interventions.

## Data Availability

The datasets presented in this article are not readily available because The datasets generated and/or analyzed during the current study are not publicly available due to confidentiality of the participants. Data are available from the corresponding author upon reasonable request. Requests to access the datasets should be directed to khalek.cnc@gmail.com.
