# Supplementary material for: Nurses’ knowledge and practice on patient safety in tertiary medical college hospitals in Bangladesh: a cross-sectional study
Source: Front Health Serv. 2026 Oct 16;6:1907119. doi: 10.3389/frhs.2026.1907119 (PMC13719546; doi:10.3389/frhs.2026.1907119)
Supplement: Supplementary file 2 [file Datasheet2.pdf]

Supplementary Materials

Supplementary Table 1.1: Sample size calculation

We calculated our required sample size by following the Agency for Healthcare Research and Quality (AHRQ) guidelines:

AHRQ guidelines reference table [32]

Number of Providers and Staff	Minimum Sample Size*	Expected Number of Responses (Assuming a 50% Response Rate)
500 (or fewer)	500 – a census of all providers and staff	250 (or fewer)
501–699	500	250
700–1,299	600	300
1,300–3,999	700	350
4,000 or more	750	375

*The sample size is based on three assumptions: simple random or systematic random sampling, a response rate of 50 percent, and a confidence interval of +/- 5 percent.

Supplementary Table 1.2: Enrolment of samples from eight different hospitals

Name of the hospitals	Total number of nurses in each hospital	Minimum sample required as per AHRQ guidelines	Actual sample collected by hospitals
Dhaka Medical College Hospital	2525	700	1119
Chattogram Medical College Hospital	1166	600	623
Rajshahi Medical College Hospital	1178	600	816
Mymensingh Medical College Hospital	1063	600	630
Khulna Medical College Hospital	475	475	354
Sher-e-Bangla Medical College Hospital	929	500	529
Rangpur Medical College and Hospital	992	600	503
Sylhet MAG Osmani Medical College Hospital	579	500	429
Total	8,907	4,575	5,003

Supplementary Table 2: Knowledge and practice level among the nurses working across eight hospitals presented as percentage (n=4982)

Level of knowledge (10 items score 0-10) and practice (10 items score 0-50)	DMCH n=1114	CMCH n=620	RMCH n=813	MMCH n=627	KMCH n=353	SBMCH n=527	RpMCH n=501	SMCH n=427
	%	%	%	%	%	%	%	%
High knowledge score > 5.44 (above the mean)	63.2	40.2	43.9	59.6	51.6	39.3	47.7	52.0
Low knowledge score ≤ 5.44 (≤ mean)	36.8	59.8	56.1	40.4	48.4	60.7	52.3	48.0
High practice score > 28.92 (above the mean)	56.2	43.7	44.8	35.2	25.8	36.8	45.1	70.0
Low practice score ≤ 28.92 (≤ mean)	43.8	56.3	55.2	64.8	74.2	63.2	54.9	30.0

Footnote: Knowledge (10 items, correct/incorrect, score 0-10) and practice (10 items, 5-point Likert, score 0-50) categorized by mean: >median = high score; ≤mean = low score.

DMCH: Dhaka Medical College Hospital; CMCH: Chattogram Medical College Hospital; RMCH: Rajshahi Medical College Hospital; MMCH: Mymensingh Medical College Hospital. KMCH: Khulna Medical College Hospital; SBMCH: Sher-e-Bangla Medical College Hospital; RpMCH: Rangpur Medical College and Hospital; SMCH: Sylhet MAG Osmani Medical College Hospital

Supplementary Table 3: Item-wise knowledge on patient safety based on correct answer across 8 hospitals' responses by nurses (n=4982) presented as percentage

Items	Knowledge of patient safety items (score-0-10)	DMCH n=1114	CMCH n=620	RMCH n=813	MMCH n=627	KMCH n=353	SBMCH n=527	RpMCH n=501	SMCH n=427
		%	%	%	%	%	%	%	%
1	Do you know about your hospital incident (adverse events) reporting system?	76.8	66.8	69.5	83.4	84.4	65.8	72.7	73.1
2	Which reporting method is used for reporting adverse events in your hospital?	13.3	12.4	10.2	6.2	3.4	5.5	12.4	9.4
3	Do you know about the 'National Hospital Policy and Procedure' for adverse event/incident reporting system?	63.5	52.3	46.5	57.7	59.8	43.5	46.1	48.5
4	Do you know 'Hospital Patient Safety Quality Improvement Team' exists in your hospital?	83.4	47.6	62.6	63.8	70.0	48.0	52.7	64.9
5	Which should be reported as an adverse event/incident?	61.3	37.6	56.6	51.8	37.7	34.2	43.7	52.0
6	Recommended hand hygiene times according to the WHO, EXCEPT	56.5	55.3	54.5	62.8	75.9	66.4	57.1	68.6
7	What is the appropriate color code for disposing of infectious waste?	77.3	64.2	82.3	75.0	73.4	70.6	53.9	68.9
8	All are Rights of Medication Administration, EXCEPT	69.5	57.3	58.7	68.1	57.5	70.8	68.1	72.4
9	Which is NOT considered an adverse event in a hospital?	51.5	6.3	48.3	66.3	53.5	57.5	62.7	56.7
10	What is the correct method for disposing of needles after use?	42.8	43.4	32.5	41.5	42.2	35.3	41.3	52.5
10 items	Mean (Median)	5.96 (6)	4.94 (5)	5.11 (5)	5.77 (6)	5.58 (6)	4.98 (5)	5.10 (5)	5.67 (6)

Footnote: DMCH: Dhaka Medical College Hospital; CMCH: Chattogram Medical College Hospital; RMCH: Rajshahi Medical College Hospital; MMCH: Mymensingh Medical College Hospital. KMCH: Khulna Medical College Hospital; SBMCH: Sher-e-Bangla Medical College Hospital; RpMCH: Rangpur Medical College and Hospital; SMCH: Sylhet MAG Osmani Medical College Hospital

Supplementary Table 4.1 – 4.8: Item-wise practice on patient safety across 8 hospitals responses by nurses (n=4982) presented as percentage

4.1 Item-wise practice on patient safety at Dhaka Medical College Hospital (DMCH) (n=1114) presented as percentage

Items of Practice on patient safety	Never %	Rarely %	Sometimes %	Most of time %	Always %	Mean (median)	Mean (median)
Do you use the Incident Reporting Form (Developed by Quality Improvement Secretariat-QIS) to report adverse events/incidents?	45.9	26.5	20.8	3.8	3.1	1.92 (2)	29.93 (30)
Do you verify patient identity using name, date of birth, and hospital registration number?	10.8	28.2	20.7	19.3	21.0	3.12 (3)	
Do you double-check medication labels and doses with your colleague before administration?	11.8	20.7	29.9	21.6	15.9	3.09 (3)	
Do you practice standard precautions, such as handwashing before and after any procedure?	7.3	21.9	27.7	29.4	13.6	3.20 (3)	
Do you use a standardized communication method like SBAR to report patient condition to physicians?	22.9	32.8	26.0	13.5	4.8	2.45 (2)	
Do you clearly and concisely share critical patient information during shift changes?	5.9	16.6	25.6	32.1	19.7	3.43 (4)	
Do you use a standard checklist while performing high-risk procedures?	11.3	15.4	24.8	30.1	18.5	3.29 (3)	
Do you report medication errors if it occurs?	7.5	18.2	34.6	25.1	14.5	3.21 (3)	
Do you check a patient's allergy history before administering medications?	8.9	21.2	35.8	23.7	10.4	3.06 (3)	
If a patient falls from bed, do you report it as an accident?	8.4	18.5	33.4	26.1	13.6	3.183)	

4.2 Item-wise practice on patient safety at Chattogram Medical College Hospital (CMCH) (n=620) presented as percentage

Items of Practice on patient safety	Never	Rarely	Sometimes	Most of time	Always	Mean (median)	Mean (median)
	%	%	%	%	%		
Do you use the Incident Reporting Form (Develop by Quality Improvement Secretariate-QIS) to report adverse events/incidents?	53.9	28.5	13.1	2.9	1.6	1.7 (1)	29.52 (27)
Do you verify patient identity using name, date of birth, and hospital registration number?	1.6	31.8	12.3	26.6	27.7	3.47 (4)	
Do you double-check medication labels and doses with your colleague before administration?	2.7	29.7	33.7	15.0	18.9	3.18 (3)	
Do you practice standard precautions, such as handwashing before and after any procedure?	2.9	30.2	37.6	12.1	17.3	3.11 (3)	
Do you use a standardized communication method like SBAR to report patient condition to physicians?	14.0	33.2	30.3	12.7	9.7	3.11 (3)	
Do you clearly and concisely share critical patient information during shift changes?	2.6	32.9	30.8	15.8	17.9	2.71(3)	
Do you use a standard checklist while performing high-risk procedures?	2.7	34.7	34.0	13.7	14.8	3.14 (3)	
Do you report medication errors if it occurs?	1.8	33.7	31.6	15.0	17.9	3.03 (3)	
Do you check a patient's allergy history before administering medications?	4.4	33.5	34.2	17.6	10.3	2.96 (3)	
If a patient falls from bed, do you report it as an accident?	2.1	34.7	28.7	19.8	14.8	3.11 (3)	

4.3 Item-wise practice on patient safety at Rajshahi Medical College Hospital, RMCH, (n=813) presented as percentage

**Items of Practice on patient safety**	**Never**	**Rarely**	**Sometimes**	**Most of time**	**Always**	**Mean (median)**	**Mean (median)**
	**%**	**%**	**%**	**%**	**%**		
Do you use the Incident Reporting Form (Develop by Quality Improvement Secretariate-QIS) to report adverse events/incidents?	40.1	32.7	20.4	5.5	1.2	1.95 (2)	28.45 (28)
Do you verify patient identity using name, date of birth, and hospital registration number?	2.0	37.8	28.8	20.8	11.4	3.01(3)	
Do you double-check medication labels and doses with your colleague before administration?	3.6	24.5	41.2	24.5	6.3	3.05 (3)	
Do you practice standard precautions, such as handwashing before and after any procedure?	2.2	26.8	39.9	27.3	3.8	3.04 (3)	
Do you use a standardized communication method like SBAR to report patient condition to physicians?	12.3	32.0	34.2	19.7	1.8	2.67 (3)	
Do you clearly and concisely share critical patient information during shift changes?	1.7	26.8	44.9	20.9	5.8	3.02 (3)	
Do you use a standard checklist while performing high-risk procedures?	3.8	28.3	41.0	22.1	4.8	2.96 (3)	
Do you report medication errors if it occurs?	3.3	29.2	35.7	28.5	3.3	2.99 (3)	
Do you check a patient's allergy history before administering medications?	4.9	30.5	37.3	25.7	1.6	2.89 (3)	
If a patient falls from bed, do you report it as an accident?	4.1	31.0	41.7	19.7	3.6	2.88 (3)	

4.4 Item-wise practice on patient safety at Mymensingh Medical College Hospital (MMCH) (n=627) presented as percentage

Items of Practice on patient safety	Never	Rarely	Sometimes	Most of time	Always	Mean (median)	Mean (median)
	%	%	%	%	%		
Do you use the Incident Reporting Form (Develop by Quality Improvement Secretariate-QIS) to report adverse events/incidents?	47.0	26.8	14.7	6.7	4.8	1.95 (2)	28.16 (25)
Do you verify patient identity using name, date of birth, and hospital registration number?	15.2	21.9	20.3	20.9	21.9	3.12 (3)	
Do you double-check medication labels and doses with your colleague before administration?	17.4	21.7	29.3	8.6	23.0	2.98 (3)	
Do you practice standard precautions, such as handwashing before and after any procedure?	12.4	30.9	24.9	12.6	19.1	2.95 (3)	
Do you use a standardized communication method like SBAR to report patient condition to physicians?	40.2	21.7	18.8	11.8	7.5	2.25 (2)	
Do you clearly and concisely share critical patient information during shift changes?	12.1	24.1	23.3	18.7	21.7	3.14 (3)	
Do you use a standard checklist while performing high-risk procedures?	10.2	27.1	27.1	16.1	19.5	3.13 (3)	
Do you report medication errors if it occurs?	10.2	27.1	27.1	16.1	19.5	3.07 (3)	
Do you check a patient's allergy history before administering medications?	10.7	25.2	41.5	13.6	9.1	2.85 (3)	
If a patient falls from bed, do you report it as an accident?	21.9	28.1	22.2	12.9	15.0	2.71 (3)	

4.5 Item-wise practice on patient safety at Khulna Medical College Hospital (KMCH) (n=353) presented as percentage

Items of Practice on patient safety	Never	Rarely	Sometimes	Most of time	Always	Mean (median)	Mean (median)
	%	%	%	%	%		
Do you use the Incident Reporting Form (Develop by Quality Improvement Secretariate-QIS) to report adverse events/incidents?	63.2	23.8	6.2	4.8	2.0	1.96 (2)	
Do you verify patient identity using name, date of birth, and hospital registration number?	6.5	45.9	30.3	9.3	7.9	3.12(3)	26.11 (25)
Do you double-check medication labels and doses with your colleague before administration?	26.6	21.8	37.4	6.2	7.9	2.98 (3)	
Do you practice standard precautions, such as handwashing before and after any procedure?	12.7	20.7	43.6	15.9	7.1	2.95 (3)	
Do you use a standardized communication method like SBAR to report patient condition to physicians?	22.1	25.5	33.4	16.1	2.8	2.25 (2)	
Do you clearly and concisely share critical patient information during shift changes?	12.5	30.6	35.1	15.0	6.8	3.14 (3)	
Do you use a standard checklist while performing high-risk procedures?	15.6	20.4	42.2	13.6	8.2	3.13 (3)	
Do you report medication errors if it occurs?	9.1	27.8	33.7	21.8	7.6	3.07 (3)	
Do you check a patient's allergy history before administering medications?	9.1	28.3	31.7	28.0	2.8	2.85 (3)	
If a patient falls from bed, do you report it as an accident?	6.5	31.7	49.0	7.6	5.1	2.71 (3)	

4.6 Item-wise practice on patient safety at Sher-e-Bangla Medical College Hospital, SBMCH, (n=527) presented as percentage

Items of Practice on patient safety	Never	Rarely	Sometimes	Most of time	Alwa ys	Mean (median)	Mean (median)
	%	%	%	%	%		
Do you use the Incident Reporting Form (Develop by Quality Improvement Secretariate-QIS) to report adverse events/incidents?	62.8	20.5	8.9	6.1	1.7	1.63(1)	26.81 (26)
Do you verify patient identity using name, date of birth, and hospital registration number?	3.0	29.8	16.3	33.6	17.3	3.32 (4)	
Do you double-check medication labels and doses with your colleague before administration?	8.9	40.2	28.3	10.6	12.0	2.76 (3)	
Do you practice standard precautions, such as handwashing before and after any procedure?	3.4	40.0	27.5	23.9	5.1	2.87 (3)	
Do you use a standardized communication method like SBAR to report patient condition to physicians?	42.9	26.0	14.6	13.3	3.2	2.08 (2)	
Do you clearly and concisely share critical patient information during shift changes?	3.8	23.9	23.0	37.6	11.8	3.30 (3)	
Do you use a standard checklist while performing high-risk procedures?	11.6	32.6	21.1	27.3	7.4	2.86 (3)	
Do you report medication errors if it occurs?	16.7	36.1	24.1	17.1	6.1	2.60 (2)	
Do you check a patient's allergy history before administering medications?	7.6	15.6	51.8	22.4	2.7	2.97 (3)	
If a patient falls from bed, do you report it as an accident?	23.9	32.3	28.1	10.4	5.3	2.41 (2)	

4.7 Item-wise practice on patient safety at Rangpur Medical College and Hospital (RpMCH) (n=501) presented as percentage

Items of Practice on patient safety	Never %	Rarely %	Sometimes %	Most of time %	Always %	Mean (median)	Mean (median)
Do you use the Incident Reporting Form (Develop by Quality Improvement Secretariate-QIS) to report adverse events/incidents?	40.9	36.5	17.2	3.0	2.4	1.89 (2)	29.26 (28)
Do you verify patient identity using name, date of birth, and hospital registration number?	2.4	22.4	33.3	23.8	18.2	3.33 (3)	
Do you double-check medication labels and doses with your colleague before administration?	5.4	20.2	33.7	26.9	13.8	3.24 (3)	
Do you practice standard precautions, such as handwashing before and after any procedure?	3.2	26.7	39.1	19.6	11.4	3.09 (3)	
Do you use a standardized communication method like SBAR to report patient condition to physicians?	35.5	19.6	26.1	11.8	7.0	2.35 (2)	
Do you clearly and concisely share critical patient information during shift changes?	4.6	18.2	35.5	31.1	10.6	3.25 (3)	
Do you use a standard checklist while performing high-risk procedures?	4.6	21.4	38.5	27.1	8.4	3.13 (3)	
Do you report medication errors if it occurs?	3.4	32.1	35.3	19.2	10.0	3.0 (3)	
Do you check a patient's allergy history before administering medications?	5.6	27.7	37.1	25.3	4.2	2.95 (3)	
If a patient falls from bed, do you report it as an accident?	3.4	26.9	41.7	19.4	8.6	3.03 (3)	

4.8 Item-wise practice on patient safety at Sylhet MAG Osmani Medical College Hospital (SMCH) (n=427) presented as percentage

Items of Practice on patient safety	Never	Rarely	Sometimes	Most of time	Always	Mean (median)	Mean (median)
	%	%	%	%	%		
Do you use the Incident Reporting Form (Develop by Quality Improvement Secretariate-QIS) to report adverse events/incidents?	45.7	35.6	12.6	4.4	1.6	1.81 (2)	31.85 (31)
Do you verify patient identity using name, date of birth, and hospital registration number?	1.9	8.2	9.8	40.0	40.0	4.08 (4)	
Do you double-check medication labels and doses with your colleague before administration?	1.9	8.2	43.1	27.9	19.0	3.54 (3)	
Do you practice standard precautions, such as handwashing before and after any procedure?	0.7	24.6	46.4	16.6	11.7	3.14 (3)	
Do you use a standardized communication method like SBAR to report patient condition to physicians?	32.3	29.0	23.2	8.2	7.3	2.29 (2)	
Do you clearly and concisely share critical patient information during shift changes?	0.7	7.0	29.5	34.7	28.1	3.82 (4)	
Do you use a standard checklist while performing high-risk procedures?	2.1	12.9	41.0	30.0	14.1	3.41 (3)	
Do you report medication errors if it occurs?	0.7	17.6	35.4	33.7	12.6	3.40 (3)	
Do you check a patient's allergy history before administering medications?	2.3	22.5	49.9	18.3	7.0	3.05 (3)	
If a patient falls from bed, do you report it as an accident?	3.3	13.1	48.5	19.7	15.5	3.31 (3)	

Supplemental Table 5: Comparison of patient safety culture by positive response rate among nurses across eight hospitals (n = 4982)

Dimensions: (items number)	DMCH (n=1114) %	CMCH (n=620) %	RMCH (n=813) %	MMCH (n=627) %	KMCH (n=353) %	SBMCH (n=527) %	RpMCH (n=501) %	SOMCH (n=427) %
D1: Teamwork (3)	79	76	77	81	83	79	76	77
D2: Staffing and work pace (4)	40	36	35	42	35	36	35	33
D3: Organizational learning-continuous improvement (3)	77	70	77	78	81	76	70	72
D4: Response to error (4)	55	50	56	52	65	48	50	57
D5: Supervisor support for patient safety (3)	75	69	72	75	85	60	63	60
D6: Communication error (3)	84	65	70	86	92	57	53	62
D7: Communication openness (4)	60	50	51	62	73	53	45	45
D8: Reporting patient safety events (2)	42	17	11	17	42	4	14	22
D9: Hospital management support for patient safety (3)	71	54	63	68	62	57	52	57
D10: Handoffs and information exchange (3)	42	39	37	41	30	37	41	32
Patient safety culture (32) by PRR	63	53	55	60	65	51	50	52

Footnote: DMCH: Dhaka Medical College Hospital; CMCH: Chattogram Medical College Hospital; RMCH: Rajshahi Medical College Hospital; MMCH: Mymensingh Medical College Hospital. KMCH: Khulna Medical College Hospital; SBMCH: Sher-e-Bangla Medical College Hospital; RpMCH: Rangpur Medical College and Hospital; SMCH: Sylhet MAG Osmani Medical College Hospital. Note: formula to calculate PRR

PRR calculation: Composite-level scores for the 10-dimension were calculated using the AHRQ formula: percent positive responses (PRR), positive responses divided by total items per dimension (34), (30). The dimensions having a PRR ≥75% were categorized as areas of strength (developed), 50–74% as potential for improvement (poorly developed) and <50% as areas of weakness (underdeveloped)

Supplemental Table 6: Patient safety event reporting and safety rating by nurses across 8 hospitals (n = 4982) presented as percentage

Variables	Categories	DMCH (n=1114)	CMCH (n=620)	RMCH (n=813)	MMCH (n=627)	KMCH (n=353)	SBMCH (n=527)	RpMCH (n=501)	SOMCH (n=427)
		%	%	%	%	%	%	%	%
Number of events reported (last 12 months)	None	21.4	28.5	34.2	19.8	39.1	24.3	34.9	36.3
	1–2	37.6	39.8	40.8	55.7	38	57.7	44.1	39.6
	3–5	21.5	23.7	18.5	19.3	11.3	16.3	16	16.4
	6–10	10.4	6.5	4.7	3.5	3.7	1.1	4.2	5.2
	11 or more	9.1	1.5	1.8	1.8	7.9	0.6	0.8	2.6
Patient safety rating	Poor	2.2	1.8	2.2	2.7	1.4	11.4	4.6	2.8
	Fair	18.9	45.2	33.8	25.4	19	34.7	27.9	39.8
	Good	42	41	50.8	57.9	42.8	41.7	55.7	39.8
	Very good	27	9.2	9.7	8.6	15.3	11	10	13.3
	Excellent	9.9	2.9	3.4	5.4	21.5	1.1	1.8	4.2

Footnote DMCH: Dhaka Medical College Hospital; CMCH: Chattogram Medical College Hospital; RMCH: Rajshahi Medical College Hospital; MMCH: Mymensingh Medical College Hospital. KMCH: Khulna Medical College Hospital; SBMCH: Sher-e-Bangla Medical College Hospital; RpMCH: Rangpur Medical College and Hospital; SMCH: Sylhet MAG Osmani Medical College Hospital. Note: formula to calculate PRR
