# Supplementary material for: Nurses’ knowledge and practice on patient safety in tertiary medical college hospitals in Bangladesh: a cross-sectional study
Source: Front Health Serv. 2026 Oct 16;6:1907119. doi: 10.3389/frhs.2026.1907119 (PMC13719546; doi:10.3389/frhs.2026.1907119)
Supplement: Supplementary file 4 [file Table1.docx]

Supplementary Material

**1.1 Sample size calculation**: **Supplementary Table 1**

We calculated our required sample size by following the Agency for Healthcare Research and Quality (AHRQ) guidelines:

**AHRQ guidelines reference table [31]**

| **Number of Providers and Staff** | **Minimum Sample Size*** | **Expected Number of Responses (Assuming a 50% Response Rate)** |
| --- | --- | --- |
| 500 (or fewer) | 500 – a census of all providers and staff | 250 (or fewer) |
| 501–699 | 500 | 250 |
| 700–1,299 | 600 | 300 |
| 1,300–3,999 | 700 | 350 |
| 4,000 or more | 750 | 375 |

*The sample size is based on three assumptions: simple random or systematic random sampling, a response rate of 50 percent, and a confidence interval of +/- 5 percent.

**1.2 Enrolment of samples from eight different hospitals**

| **Name of the hospitals** | **Total number of nurses in each hospital** | **Minimum sample required as per AHRQ guidelines** | **Actual sample collected by hospitals** |
| --- | --- | --- | --- |
| Dhaka Medical College Hospital | 2525 | 700 | 1119 |
| Chattrogram Medical College Hospital | 1166 | 600 | 623 |
| Rajshahi Medical College Hospital | 1178 | 600 | 816 |
| Mymensingh Medical College Hospital | 1063 | 600 | 630 |
| Khulna Medical College Hospital | 475 | 475 | 354 |
| Sher-e-Bangla Medical College Hospital | 929 | 500 | 529 |
| Rangpur Medical College and Hospital | 992 | 600 | 503 |
| Sylhet MAG Osmani Medical College Hospital | 579 | 500 | 429 |
| **Total** | **8,907** | **4,575** | **5,003** |

**Supplementary Table 2**

## 2.1 Knowledge and practice level among the nurses working across eight hospitals presented as percentage (N=4982)

| **Level of knowledge (10 items score 0-10) and practice (10 items score 0-50)** | **DMCH n=1114** | **CMCH n=620** | **RMCH n=813** | **MMCH n=627** | **KMCH n=353** | **SBMCH n=527** | **RpMCH n=501** | **SMCH n=427** |
| --- | --- | --- | --- | --- | --- | --- | --- | --- |
|  | **%** | **%** | **%** | **%** | **%** | **%** | **%** | **%** |
| **Poor knowledge ≤ 6 (median=6)** | 59 | 83.2 | 76.3 | 61.1 | 69.4 | 78.6 | 77 | 59.5 |
| **Good knowledge > 6** | 41 | 16.8 | 23.7 | 38.9 | 30.6 | 21.4 | 23 | 40.5 |
| **Poor practice ≤ 28 (median=28)** | 43.8 | 56.3 | 55.2 | 64.8 | 74.2 | 63.2 | 54.9 | 30 |
| **Good practice > 28** | 56.2 | 43.7 | 44.8 | 35.2 | 25.8 | 36.8 | 45.1 | 70 |

**Table footnote**: Knowledge (10 items, correct/incorrect, score 0-10) and practice (10 items, 5-point Likert, score 0-50) categorized by median split: >median = good; ≤median = poor.

DMCH: Dhaka Medical College Hospital; CMCH: Chattogram Medical College Hospital; RMCH: Rajshahi Medical College Hospital; MMCH: Mymensingh Medical College Hospital. KMCH: Khulna Medical College Hospital; SBMCH: Sher-e-Bangla Medical College Hospital; RpMCH: Rangpur Medical College and Hospital; SMCH: Sylhet MAG Osmani Medical College Hospital

**Supplementary Table 3**

## 3.1 Item wise knowledge on patient safety based on correct answer across 8 hospitals responses by nurses (N=4982) presented as percentage

| **Items** | **Knowledge of patient safety items (score-0-10)** | **DMCH n=1114** | **CMCH n=620** | **RMCH n=813** | **MMCH n=627** | **KMCH n=353** | **SBMCH n=527** | **RpMCH n=501** | **SMCH n=427** |
| --- | --- | --- | --- | --- | --- | --- | --- | --- | --- |
|  |  | **%** | **%** | **%** | **%** | **%** | **%** | **%** | **%** |
| 1 | Do you know about your hospital incident (adverse events) reporting system? | 76.8 | 66.8 | 69.5 | 83.4 | 84.4 | 65.8 | 72.7 | 73.1 |
| 2 | Which reporting method is used for reporting adverse events in your hospital? | 13.3 | 12.4 | 10.2 | 6.2 | 3.4 | 5.5 | 12.4 | 9.4 |
| 3 | Do you know about the 'National Hospital Policy and Procedure' for adverse event/incident reporting system? | 63.5 | 52.3 | 46.5 | 57.7 | 59.8 | 43.5 | 46.1 | 48.5 |
| 4 | Do you know 'Hospital Patient Safety Quality Improvement Team' exists in your hospital? | 83.4 | 47.6 | 62.6 | 63.8 | 70.0 | 48.0 | 52.7 | 64.9 |
| 5 | Which should be reported as an adverse event/incident? | 61.3 | 37.6 | 56.6 | 51.8 | 37.7 | 34.2 | 43.7 | 52.0 |
| 6 | Recommended hand hygiene times according to the WHO, EXCEPT | 56.5 | 55.3 | 54.5 | 62.8 | 75.9 | 66.4 | 57.1 | 68.6 |
| 7 | What is the appropriate color code for disposing of infectious waste? | 77.3 | 64.2 | 82.3 | 75.0 | 73.4 | 70.6 | 53.9 | 68.9 |
| 8 | All are Rights of Medication Administration, EXCEPT | 69.5 | 57.3 | 58.7 | 68.1 | 57.5 | 70.8 | 68.1 | 72.4 |
| 9 | Which is NOT considered an adverse event in a hospital? | 51.5 | 6.3 | 48.3 | 66.3 | 53.5 | 57.5 | 62.7 | 56.7 |
| 10 | What is the correct method for disposing of needles after use? | 42.8 | 43.4 | 32.5 | 41.5 | 42.2 | 35.3 | 41.3 | 52.5 |
| 10 items | **Mean**  **(Median)** | **5.96**  **(6)** | **4.94**  **(5)** | **5.11**  **(5)** | **5.77**  **(6)** | **5.58**  **(6)** | **4.98**  **(5)** | **5.10**  **(5)** | **5.67**  **(6)** |

**Table footnote**: DMCH: Dhaka Medical College Hospital; CMCH: Chattogram Medical College Hospital; RMCH: Rajshahi Medical College Hospital; MMCH: Mymensingh Medical College Hospital. KMCH: Khulna Medical College Hospital; SBMCH: Sher-e-Bangla Medical College Hospital; RpMCH: Rangpur Medical College and Hospital; SMCH: Sylhet MAG Osmani Medical College Hospital

**Supplementary Table 4**

## 4 Item wise practice on patient safety across 8 hospitals responses by nurses (N=4982) presented as percentage

## 4.1 Item wise practice on patient safety at Dhaka Medical College Hospital, DMCH, (n=1114) presented as percentage

| **Items of Practice on patient safety** | **Never** | **Rarely** | **Sometimes** | **Most of time** | **Always** | **Mean (median)** | **Mean (median)** |
| --- | --- | --- | --- | --- | --- | --- | --- |
|  | **%** | **%** | **%** | **%** | **%** |  |  |
| Do you use the Incident Reporting Form (Develop by Quality Improvement Secretariate-QIS) to report adverse events/incidents? | 45.9 | 26.5 | 20.8 | 3.8 | 3.1 | 1.92 (2) | **29.93 (30)** |
| Do you verify patient identity using name, date of birth, and hospital registration number? | 10.8 | 28.2 | 20.7 | 19.3 | 21.0 | 3.12 (3) |  |
| Do you double-check medication labels and doses with your colleague before administration? | 11.8 | 20.7 | 29.9 | 21.6 | 15.9 | 3.09 (3) |  |
| Do you practice standard precautions, such as handwashing before and after any procedure? | 7.3 | 21.9 | 27.7 | 29.4 | 13.6 | 3.20 (3) |  |
| Do you use a standardized communication method like SBAR to report patient condition to physicians? | 22.9 | 32.8 | 26.0 | 13.5 | 4.8 | 2.45 (2) |  |
| Do you clearly and concisely share critical patient information during shift changes? | 5.9 | 16.6 | 25.6 | 32.1 | 19.7 | 3.43 (4) |  |
| Do you use a standard checklist while performing high-risk procedures? | 11.3 | 15.4 | 24.8 | 30.1 | 18.5 | 3.29 (3) |  |
| Do you report medication errors if it occurs? | 7.5 | 18.2 | 34.6 | 25.1 | 14.5 | 3.21 (3) |  |
| Do you check a patient's allergy history before administering medications? | 8.9 | 21.2 | 35.8 | 23.7 | 10.4 | 3.06 (3) |  |
| If a patient falls from bed, do you report it as an accident? | 8.4 | 18.5 | 33.4 | 26.1 | 13.6 | 3.18 3) |  |

## 4.2 Item wise practice on patient safety at Chattogram Medical College Hospital CMCH, (n=620) presented as percentage

| **Items of Practice on patient safety** | **Never** | **Rarely** | **Sometimes** | **Most of time** | **Always** | **Mean (median)** | **Mean (median)** |
| --- | --- | --- | --- | --- | --- | --- | --- |
|  | **%** | **%** | **%** | **%** | **%** |  |  |
| Do you use the Incident Reporting Form (Develop by Quality Improvement Secretariate-QIS) to report adverse events/incidents? | 53.9 | 28.5 | 13.1 | 2.9 | 1.6 | 1.7 (1) | **29.52 (27)** |
| Do you verify patient identity using name, date of birth, and hospital registration number? | 1.6 | 31.8 | 12.3 | 26.6 | 27.7 | 3.47 (4) |  |
| Do you double-check medication labels and doses with your colleague before administration? | 2.7 | 29.7 | 33.7 | 15.0 | 18.9 | 3.18 (3) |  |
| Do you practice standard precautions, such as handwashing before and after any procedure? | 2.9 | 30.2 | 37.6 | 12.1 | 17.3 | 3.11 (3) |  |
| Do you use a standardized communication method like SBAR to report patient condition to physicians? | 14.0 | 33.2 | 30.3 | 12.7 | 9.7 | 3.11 (3) |  |
| Do you clearly and concisely share critical patient information during shift changes? | 2.6 | 32.9 | 30.8 | 15.8 | 17.9 | 2.71(3) |  |
| Do you use a standard checklist while performing high-risk procedures? | 2.7 | 34.7 | 34.0 | 13.7 | 14.8 | 3.14 (3) |  |
| Do you report medication errors if it occurs? | 1.8 | 33.7 | 31.6 | 15.0 | 17.9 | 3.03 (3) |  |
| Do you check a patient's allergy history before administering medications? | 4.4 | 33.5 | 34.2 | 17.6 | 10.3 | 2.96 (3) |  |
| If a patient falls from bed, do you report it as an accident? | 2.1 | 34.7 | 28.7 | 19.8 | 14.8 | 3.11 (3) |  |

**4.3 Item wise practice on patient safety at Rajshahi Medical College Hospital, RMCH, (n=813) presented as percentage**

| **Items of Practice on patient safety** | **Never** | **Rarely** | **Sometimes** | **Most of time** | **Always** | **Mean (median)** | **Mean (median)** |
| --- | --- | --- | --- | --- | --- | --- | --- |
|  | **%** | **%** | **%** | **%** | **%** |  |  |
| Do you use the Incident Reporting Form (Develop by Quality Improvement Secretariate-QIS) to report adverse events/incidents? | 40.1 | 32.7 | 20.4 | 5.5 | 1.2 | 1.95 (2) | **28.45(28**) |
| Do you verify patient identity using name, date of birth, and hospital registration number? | 2.0 | 37.8 | 28.8 | 20.8 | 11.4 | 3.01(3) |  |
| Do you double-check medication labels and doses with your colleague before administration? | 3.6 | 24.5 | 41.2 | 24.5 | 6.3 | 3.05 (3) |  |
| Do you practice standard precautions, such as handwashing before and after any procedure? | 2.2 | 26.8 | 39.9 | 27.3 | 3.8 | 3.04 (3) |  |
| Do you use a standardized communication method like SBAR to report patient condition to physicians? | 12.3 | 32.0 | 34.2 | 19.7 | 1.8 | 2.67 (3) |  |
| Do you clearly and concisely share critical patient information during shift changes? | 1.7 | 26.8 | 44.9 | 20.9 | 5.8 | 3.02 (3) |  |
| Do you use a standard checklist while performing high-risk procedures? | 3.8 | 28.3 | 41.0 | 22.1 | 4.8 | 2.96 (3) |  |
| Do you report medication errors if it occurs? | 3.3 | 29.2 | 35.7 | 28.5 | 3.3 | 2.99 (3) |  |
| Do you check a patient's allergy history before administering medications? | 4.9 | 30.5 | 37.3 | 25.7 | 1.6 | 2.89 (3) |  |
| If a patient falls from bed, do you report it as an accident? | 4.1 | 31.0 | 41.7 | 19.7 | 3.6 | 2.88 (3) |  |

**4.4 Item wise practice on patient safety at Mymensingh Medical College Hospital, MMCH, (n=627) presented as percentage**

| **Items of Practice on patient safety** | **Never** | **Rarely** | **Sometimes** | **Most of time** | **Always** | **Mean (median)** | **Mean (median)** |
| --- | --- | --- | --- | --- | --- | --- | --- |
|  | **%** | **%** | **%** | **%** | **%** |  |  |
| Do you use the Incident Reporting Form (Develop by Quality Improvement Secretariate-QIS) to report adverse events/incidents? | 47.0 | 26.8 | 14.7 | 6.7 | 4.8 | 1.95 (2) | **28.16 (25)** |
| Do you verify patient identity using name, date of birth, and hospital registration number? | 15.2 | 21.9 | 20.3 | 20.9 | 21.9 | 3.12 (3) |  |
| Do you double-check medication labels and doses with your colleague before administration? | 17.4 | 21.7 | 29.3 | 8.6 | 23.0 | 2.98 (3) |  |
| Do you practice standard precautions, such as handwashing before and after any procedure? | 12.4 | 30.9 | 24.9 | 12.6 | 19.1 | 2.95 (3) |  |
| Do you use a standardized communication method like SBAR to report patient condition to physicians? | 40.2 | 21.7 | 18.8 | 11.8 | 7.5 | 2.25 (2) |  |
| Do you clearly and concisely share critical patient information during shift changes? | 12.1 | 24.1 | 23.3 | 18.7 | 21.7 | 3.14 (3) |  |
| Do you use a standard checklist while performing high-risk procedures? | 10.2 | 27.1 | 27.1 | 16.1 | 19.5 | 3.13 (3) |  |
| Do you report medication errors if it occurs? | 10.2 | 27.1 | 27.1 | 16.1 | 19.5 | 3.07 (3) |  |
| Do you check a patient's allergy history before administering medications? | 10.7 | 25.2 | 41.5 | 13.6 | 9.1 | 2.85 (3) |  |
| If a patient falls from bed, do you report it as an accident? | 21.9 | 28.1 | 22.2 | 12.9 | 15.0 | 2.71 (3) |  |

**4.5 Item wise practice on patient safety at Khulna Medical College Hospital, KMCH, (n=353) presented as percentage**

| **Items of Practice on patient safety** | **Never** | **Rarely** | **Sometimes** | **Most of time** | **Always** | **Mean (median)** | **Mean (median)** |
| --- | --- | --- | --- | --- | --- | --- | --- |
|  | **%** | **%** | **%** | **%** | **%** |  |  |
| Do you use the Incident Reporting Form (Develop by Quality Improvement Secretariate-QIS) to report adverse events/incidents? | 63.2 | 23.8 | 6.2 | 4.8 | 2.0 | 1.96 (2) |  |
| Do you verify patient identity using name, date of birth, and hospital registration number? | 6.5 | 45.9 | 30.3 | 9.3 | 7.9 | 3.12(3) | **26.11 (25)** |
| Do you double-check medication labels and doses with your colleague before administration? | 26.6 | 21.8 | 37.4 | 6.2 | 7.9 | 2.98 (3) |  |
| Do you practice standard precautions, such as handwashing before and after any procedure? | 12.7 | 20.7 | 43.6 | 15.9 | 7.1 | 2.95 (3) |  |
| Do you use a standardized communication method like SBAR to report patient condition to physicians? | 22.1 | 25.5 | 33.4 | 16.1 | 2.8 | 2.25 (2) |  |
| Do you clearly and concisely share critical patient information during shift changes? | 12.5 | 30.6 | 35.1 | 15.0 | 6.8 | 3.14 (3) |  |
| Do you use a standard checklist while performing high-risk procedures? | 15.6 | 20.4 | 42.2 | 13.6 | 8.2 | 3.13 (3) |  |
| Do you report medication errors if it occurs? | 9.1 | 27.8 | 33.7 | 21.8 | 7.6 | 3.07 (3) |  |
| Do you check a patient's allergy history before administering medications? | 9.1 | 28.3 | 31.7 | 28.0 | 2.8 | 2.85 (3) |  |
| If a patient falls from bed, do you report it as an accident? | 6.5 | 31.7 | 49.0 | 7.6 | 5.1 | 2.71 (3) |  |

**4.6 Item wise practice on patient safety at Sher-e-Bangla Medical College Hospital, SBMCH, (n=527) presented as percentage**

| **Items of Practice on patient safety** | **Never** | **Rarely** | **Sometimes** | **Most of time** | **Always** | **Mean (median)** | **Mean (median)** |
| --- | --- | --- | --- | --- | --- | --- | --- |
|  | **%** | **%** | **%** | **%** | **%** |  |  |
| Do you use the Incident Reporting Form (Develop by Quality Improvement Secretariate-QIS) to report adverse events/incidents? | 62.8 | 20.5 | 8.9 | 6.1 | 1.7 | 1.63(1) | **26.81 (26)** |
| Do you verify patient identity using name, date of birth, and hospital registration number? | 3.0 | 29.8 | 16.3 | 33.6 | 17.3 | 3.32 (4) |  |
| Do you double-check medication labels and doses with your colleague before administration? | 8.9 | 40.2 | 28.3 | 10.6 | 12.0 | 2.76 (3) |  |
| Do you practice standard precautions, such as handwashing before and after any procedure? | 3.4 | 40.0 | 27.5 | 23.9 | 5.1 | 2.87 (3) |  |
| Do you use a standardized communication method like SBAR to report patient condition to physicians? | 42.9 | 26.0 | 14.6 | 13.3 | 3.2 | 2.08 (2) |  |
| Do you clearly and concisely share critical patient information during shift changes? | 3.8 | 23.9 | 23.0 | 37.6 | 11.8 | 3.30 (3) |  |
| Do you use a standard checklist while performing high-risk procedures? | 11.6 | 32.6 | 21.1 | 27.3 | 7.4 | 2.86 (3) |  |
| Do you report medication errors if it occurs? | 16.7 | 36.1 | 24.1 | 17.1 | 6.1 | 2.60 (2) |  |
| Do you check a patient's allergy history before administering medications? | 7.6 | 15.6 | 51.8 | 22.4 | 2.7 | 2.97 (3) |  |
| If a patient falls from bed, do you report it as an accident? | 23.9 | 32.3 | 28.1 | 10.4 | 5.3 | 2.41 (2) |  |

**4.7 Item wise practice on patient safety at Rangpur Medical College and Hospital, RpMCH, (n=501) presented as percentage**

| **Items of Practice on patient safety** | **Never** | **Rarely** | **Sometimes** | **Most of time** | **Always** | **Mean (median)** | **Mean (median)** |
| --- | --- | --- | --- | --- | --- | --- | --- |
|  | **%** | **%** | **%** | **%** | **%** |  |  |
| Do you use the Incident Reporting Form (Develop by Quality Improvement Secretariate-QIS) to report adverse events/incidents? | 40.9 | 36.5 | 17.2 | 3.0 | 2.4 | 1.89 (2) | **29.26 (28)** |
| Do you verify patient identity using name, date of birth, and hospital registration number? | 2.4 | 22.4 | 33.3 | 23.8 | 18.2 | 3.33 (3) |  |
| Do you double-check medication labels and doses with your colleague before administration? | 5.4 | 20.2 | 33.7 | 26.9 | 13.8 | 3.24 (3) |  |
| Do you practice standard precautions, such as handwashing before and after any procedure? | 3.2 | 26.7 | 39.1 | 19.6 | 11.4 | 3.09 (3) |  |
| Do you use a standardized communication method like SBAR to report patient condition to physicians? | 35.5 | 19.6 | 26.1 | 11.8 | 7.0 | 2.35 (2) |  |
| Do you clearly and concisely share critical patient information during shift changes? | 4.6 | 18.2 | 35.5 | 31.1 | 10.6 | 3.25 (3) |  |
| Do you use a standard checklist while performing high-risk procedures? | 4.6 | 21.4 | 38.5 | 27.1 | 8.4 | 3.13 (3) |  |
| Do you report medication errors if it occurs? | 3.4 | 32.1 | 35.3 | 19.2 | 10.0 | 3.0 (3) |  |
| Do you check a patient's allergy history before administering medications? | 5.6 | 27.7 | 37.1 | 25.3 | 4.2 | 2.95 (3) |  |
| If a patient falls from bed, do you report it as an accident? | 3.4 | 26.9 | 41.7 | 19.4 | 8.6 | 3.03 (3) |  |

**4.8 Item wise practice on patient safety at Sylhet MAG Osmani Medical College Hospital, SMCH, (n=427) presented as percentage**

| **Items of Practice on patient safety** | **Never** | **Rarely** | **Sometimes** | **Most of time** | **Always** | **Mean (median)** | **Mean (median)** |
| --- | --- | --- | --- | --- | --- | --- | --- |
|  | **%** | **%** | **%** | **%** | **%** |  |  |
| Do you use the Incident Reporting Form (Develop by Quality Improvement Secretariate-QIS) to report adverse events/incidents? | 45.7 | 35.6 | 12.6 | 4.4 | 1.6 | 1.81 (2) | **31.85 (31)** |
| Do you verify patient identity using name, date of birth, and hospital registration number? | 1.9 | 8.2 | 9.8 | 40.0 | 40.0 | 4.08 (4) |  |
| Do you double-check medication labels and doses with your colleague before administration? | 1.9 | 8.2 | 43.1 | 27.9 | 19.0 | 3.54 (3) |  |
| Do you practice standard precautions, such as handwashing before and after any procedure? | 0.7 | 24.6 | 46.4 | 16.6 | 11.7 | 3.14 (3) |  |
| Do you use a standardized communication method like SBAR to report patient condition to physicians? | 32.3 | 29.0 | 23.2 | 8.2 | 7.3 | 2.29 (2) |  |
| Do you clearly and concisely share critical patient information during shift changes? | 0.7 | 7.0 | 29.5 | 34.7 | 28.1 | 3.82 (4) |  |
| Do you use a standard checklist while performing high-risk procedures? | 2.1 | 12.9 | 41.0 | 30.0 | 14.1 | 3.41 (3) |  |
| Do you report medication errors if it occurs? | 0.7 | 17.6 | 35.4 | 33.7 | 12.6 | 3.40 (3) |  |
| Do you check a patient's allergy history before administering medications? | 2.3 | 22.5 | 49.9 | 18.3 | 7.0 | 3.05 (3) |  |
| If a patient falls from bed, do you report it as an accident? | 3.3 | 13.1 | 48.5 | 19.7 | 15.5 | 3.31 (3) |  |

**Supplemental Table 5.**

## 5.1 Comparison of patient safety culture by positive response rate among nurses across eight hospitals (N = 4982)

| **Dimensions: (items number)** | **DMCH (n=1114)**  **%** | **CMCH (n=620)**  **%** | **RMCH (n=813)**  **%** | **MMCH (n=627)**  **%** | **KMCH (n=353)**  **%** | **SBMCH (n=527)**  **%** | **RpMCH (n=501)**  **%** | **SOMCH (n=427)**  **%** |
| --- | --- | --- | --- | --- | --- | --- | --- | --- |
| D1: Teamwork (3) | 79 | 76 | 77 | 81 | 83 | 79 | 76 | 77 |
| D2: Staffing and work pace (4) | 40 | 36 | 35 | 42 | 35 | 36 | 35 | 33 |
| D3: Organizational learning-continuous improvement (3) | 77 | 70 | 77 | 78 | 81 | 76 | 70 | 72 |
| D4: Response to error (4) | 55 | 50 | 56 | 52 | 65 | 48 | 50 | 57 |
| D5: Supervisor support for patient safety (3) | 75 | 69 | 72 | 75 | 85 | 60 | 63 | 60 |
| D6: Communication error (3) | 84 | 65 | 70 | 86 | 92 | 57 | 53 | 62 |
| D7: Communication openness (4) | 60 | 50 | 51 | 62 | 73 | 53 | 45 | 45 |
| D8: Reporting patient safety events (2) | 42 | 17 | 11 | 17 | 42 | 4 | 14 | 22 |
| D9: Hospital management support for patient safety (3) | 71 | 54 | 63 | 68 | 62 | 57 | 52 | 57 |
| D10: Handoffs and information exchange (3) | 42 | 39 | 37 | 41 | 30 | 37 | 41 | 32 |
| **Patient safety culture (32) by PRR** | **63** | **53** | **55** | **60** | **65** | **51** | **50** | **52** |

**Table footnote**: DMCH: Dhaka Medical College Hospital; CMCH: Chattogram Medical College Hospital; RMCH: Rajshahi Medical College Hospital; MMCH: Mymensingh Medical College Hospital. KMCH: Khulna Medical College Hospital; SBMCH: Sher-e-Bangla Medical College Hospital; RpMCH: Rangpur Medical College and Hospital; SMCH: Sylhet MAG Osmani Medical College Hospital. Note: formula to calculate PRR

**PRR calculation:** Composite-level scores for the 10-dimension were calculated using the AHRQ formula: percent positive responses (PRR), positive responses divided by total items per dimension (34), (30). The dimensions having a PRR ≥75% were categorized as areas of strength (developed), 50–74% as potential for improvement (poorly developed) and <50% as areas of weakness (underdeveloped)

**Supplemental Table 6.**

## 6.1 Patient safety event reporting and safety rating by nurses across 8 hospitals (N = 4982) presented as percentage

| **Variables** | **Categories** | **DMCH (n=1114)** | **CMCH (n=620)** | **RMCH (n=813)** | **MMCH (n=627)** | **KMCH (n=353)** | **SBMCH (n=527)** | **RpMCH (n=501)** | **SOMCH (n=427)** |
| --- | --- | --- | --- | --- | --- | --- | --- | --- | --- |
|  |  | **%** | **%** | **%** | **%** | **%** | **%** | **%** | **%** |
| **Number of events reported**  **(last 12 months)** | None | 21.4 | 28.5 | 34.2 | 19.8 | 39.1 | 24.3 | 34.9 | 36.3 |
|  | 1–2 | 37.6 | 39.8 | 40.8 | 55.7 | 38 | 57.7 | 44.1 | 39.6 |
|  | 3–5 | 21.5 | 23.7 | 18.5 | 19.3 | 11.3 | 16.3 | 16 | 16.4 |
|  | 6–10 | 10.4 | 6.5 | 4.7 | 3.5 | 3.7 | 1.1 | 4.2 | 5.2 |
|  | 11 or more | 9.1 | 1.5 | 1.8 | 1.8 | 7.9 | 0.6 | 0.8 | 2.6 |
| **Patient safety rating** | Poor | 2.2 | 1.8 | 2.2 | 2.7 | 1.4 | 11.4 | 4.6 | 2.8 |
|  | Fair | 18.9 | 45.2 | 33.8 | 25.4 | 19 | 34.7 | 27.9 | 39.8 |
|  | Good | 42 | 41 | 50.8 | 57.9 | 42.8 | 41.7 | 55.7 | 39.8 |
|  | Very good | 27 | 9.2 | 9.7 | 8.6 | 15.3 | 11 | 10 | 13.3 |
|  | Excellent | 9.9 | 2.9 | 3.4 | 5.4 | 21.5 | 1.1 | 1.8 | 4.2 |

**Table footnote** DMCH: Dhaka Medical College Hospital; CMCH: Chattogram Medical College Hospital; RMCH: Rajshahi Medical College Hospital; MMCH: Mymensingh Medical College Hospital. KMCH: Khulna Medical College Hospital; SBMCH: Sher-e-Bangla Medical College Hospital; RpMCH: Rangpur Medical College and Hospital; SMCH: Sylhet MAG Osmani Medical College Hospital. Note: formula to calculate PRR
